# Sleep spindle density and morphology are resilient to post-traumatic gray matter volume loss^☆^

**DOI:** 10.1016/j.nicl.2025.103915

**Published:** 2025-11-26

**Authors:** Narges Kalantari, Véronique Daneault, Hélène Blais, Claire André, Erlan Sanchez, Jean-Marc Lina, Caroline Arbour, Danielle Gilbert, Julie Carrier, Nadia Gosselin

**Affiliations:** a Center for Advanced Research in Sleep Medicine, Hôpital du Sacré-Cœur de Montréal, Centre intégré universitaire de santé et de services sociaux du Nord de l’Île-de Montréal, Montreal, QC H4J 1C5, Canada; b Department of Psychology, Université de Montréal, Montreal, QC H2V 2S9, Canada; c Cognitive Neurology Research Unit, Sunnybrook Research Institute, Toronto, ON M4N 3M5, Canada; d Department of Electrical Engineering, École de Technologie Supérieure, Montreal, QC H3C 1K3, Canada; e Faculty of Nursing, Université de Montréal, Montreal, QC H3T 1A8, Canada; f Department of Radiology, Radiation Oncology, and Nuclear Medicine, Université de Montréal, Montreal, QC H3T 1A4, Canada; g Department of Radiology, Hôpital du Sacré-Coeur de Montréal, Centre intégré universitaire de santé et de services sociaux du Nord de l’Île-de Montréal, Montreal, QC H4J 1C5, Canada

**Keywords:** Traumatic brain injury, Cerebral gray matter, Sleep, Sleep spindles, Sigma spectral power, Electroencephalography

## Abstract

Moderate to severe traumatic brain injury (TBI) leads to gray matter volume (GMV) loss, cognitive dysfunction, and persistent sleep–wake complaints. Given the link between GMV and sleep spindles in healthy adults and the role of spindles in neural plasticity and protecting sleep against disturbances, we investigated GMV–spindle associations following TBI. In this cross-sectional study, 27 adults with chronic moderate to severe TBI (32.0 ± 12.2 years old) and 32 healthy controls (29.2 ± 11.5 years old) underwent full-night polysomnography and 3-Tesla MRI. Spindle density, amplitude, frequency, duration, and sigma spectral power (11–16 Hz) were computed. We tested GMV–spindle associations in 1) clusters with GMV loss following TBI (right and left frontotemporal and left temporal) and 2) regions previously linked to spindles in healthy adults (hippocampus, insula, cingulate, supplementary motor area, cerebellum, Heschl’s gyri, thalamus, medial prefrontal cortex, putamen, and pallidum). Multiple regression analyses were performed with Group as a moderator, controlled for age. Across all participants, higher spindle amplitude and sigma power were associated with larger GMVs in the left frontotemporal, left temporal, thalamic, and medial prefrontal regions. Faster spindle frequency was associated with larger GMV in most regions, though for the left and right frontotemporal regions and hippocampus, these associations were observed only in controls. No Group effects were found for spindle characteristics. The lack of stronger GMV–spindle associations following TBI and the absence of Group effects for spindle characteristics suggest spindles’ resilience to post-traumatic GMV loss.

## Highlights

• Sleep spindle parameters are linked to gray matter integrity in healthy adults.
• Moderate to severe traumatic brain injury results in extensive gray matter atrophy.
• Brain injury did not result in more pronounced gray matter–spindle associations.
• Spindles remained resilient to gray matter volume loss.
• Factors beyond gray matter volume may better explain spindle morphology.

## 1 Introduction

Sleep spindles are transient bursts of oscillatory activity during non-rapid eye movement (NREM) sleep that are generated through reciprocal interactions within the thalamocortical network (Clawson et al., 2016). They are proposed to protect sleep from disturbances caused by external auditory stimuli (Dang-Vu et al., 2010) and facilitate neural plasticity underlying learning and memory consolidation (Fernandez and Lüthi, 2020). Given the persistent sleep–wake disturbances (Sandsmark et al., 2017) and cognitive impairments (Rabinowitz and Levin, 2014) following traumatic brain injury (TBI), and the critical role of sleep in recovery, investigating sleep spindles is highly relevant in this population. Importantly, moderate to severe TBI leads to extensive loss of gray matter volume (GMV) across cortical and subcortical regions, most prominently in the thalamus, hippocampus, pallidum, cerebellum, and insula, with atrophy continuing into the chronic phase of injury (for a review, see Harris et al., 2019). Therefore, moderate to severe TBI provides an opportunity to study the relationship between extensive GMV loss and sleep spindle characteristics.

Some studies have tested the association between GMV and sleep spindles in healthy adults. In a study of healthy young adults, larger GMVs in the insula and auditory cortex were associated with slower spindle frequency, whereas larger GMV in the hippocampus was associated with faster spindles (Saletin et al., 2013). Other studies have compared spindles in younger and older individuals. With aging, a decrease in spindle density, amplitude, duration, and sigma power (spectral power within the spindle range, 11–16 Hz) (Carrier et al., 2001, Crowley et al., 2002, Fitzroy et al., 2021, Fogel et al., 2017, Guazzelli et al., 1986, Martin et al., 2013, Muehlroth et al., 2020, Nicolas et al., 2001, Principe and Smith, 1982, Purcell et al., 2017, Toor et al., 2023, Wei et al., 1999), and a slight increase in spindle frequency (Crowley et al., 2002, Martin et al., 2013, Nicolas et al., 2001, Principe and Smith, 1982, Wei et al., 1999) have been generally demonstrated. One study reported associations between larger GMVs in the medial prefrontal cortex, thalamus, hippocampus, and entorhinal cortex and higher spindle density across healthy young and older participants (Muehlroth et al., 2020). Interestingly, daytime nap studies have reported age-related differences in GMV–spindle associations, with generally more positive associations in younger adults than older adults (Fitzroy et al., 2021, Fogel et al., 2017, Toor et al., 2023). Specifically, as compared to older adults, larger GMVs, mainly in the cerebellum, hippocampus, and cingulate, correlated to a greater extent with longer spindle duration in younger adults (Fogel et al., 2017). In another daytime nap study, GMVs mainly in the premotor and supplementary motor area correlated more positively with spindle amplitude in younger adults and more negatively in older adults (Toor et al., 2023). Similarly, GMVs in the pallidum and putamen generally correlated positively with higher sigma spectral power in younger adults and negatively in older adults (Fitzroy et al., 2021). Overall, these studies suggest that spindles are associated with GMV, though these associations seem to be different in younger and older adults.

Due to the widespread brain atrophy secondary to moderate to severe TBI (Harris et al., 2019), this population offers a valuable opportunity to investigate the structural brain correlates of NREM sleep oscillations (Kalantari et al., 2024, Sanchez et al., 2020, Sanchez et al., 2019). However, these associations remain largely understudied. In an earlier study performed on a subset of patients from the present sample, our group found no difference in sleep spindle density, amplitude, frequency, duration, or sigma power between 23 individuals with chronic moderate to severe TBI and 27 healthy controls (Sanchez et al., 2020). However, in the TBI group only, white matter damage was associated with slower spindle frequency and, to a smaller extent, with shorter spindle duration. Building on our earlier investigation, the present study aimed to examine the link between cerebral gray matter atrophy following moderate to severe TBI and spindle density and morphology, including maximal peak-to-peak amplitude, mean frequency, and duration, as well as sigma spectral power (11–16 Hz), compared to healthy adults. Because of the extensive gray matter atrophy secondary to moderate to severe TBI that leads to a greater variability in GMV, we expected these associations to be more pronounced (i.e., stronger positive associations) in the TBI group than in healthy controls. Alternatively, if spindles are resilient to change following brain injury, their characteristics may remain stable despite GMV loss. In this case, the expected positive associations between GMV and spindles may weaken in the TBI group, as spindle characteristics would be less dependent on the changes in cerebral gray matter structure following brain injury. This study will provide a better understanding of how spindle morphology and expression relate to widespread gray matter atrophy following moderate to severe TBI.

## 2 Materials and methods

### 2.1 Participants

The details of the study protocol have been described previously (Kalantari et al., 2024, Sanchez et al., 2020, Sanchez et al., 2019). This study included 27 adults in the chronic stage of moderate to severe TBI (18–60 years; 11–39 months post-TBI). Eligibility was assessed for all moderate to severe TBI patients admitted to the Hôpital du Sacré-Cœur de Montréal between 2010 and 2016. The diagnosis of moderate to severe TBI was confirmed by a neurosurgeon based on the Glasgow Coma Scale, as well as the duration of unconsciousness and post-traumatic amnesia (National Academies, 2019). Specifically, TBI was classified as moderate if patients scored 9–12 on the Glasgow Coma Scale, experienced a loss of consciousness lasting 30 min to 24 h, and had post-traumatic amnesia lasting 24 h to 14 days. TBI was classified as severe if patients scored 3–8 on the Glasgow Coma Scale, experienced a loss of consciousness of more than 24 h, and had post-traumatic amnesia lasting more than 14 days.

Medical charts were reviewed, and eligible patients were contacted for a structural phone interview. Participants with TBI were excluded based on the following criteria: 1) history of diagnosed psychiatric, neurologic, or substance abuse disorders; 2) history of diagnosed sleep disorders prior to TBI; 3) sleep medication intake and inability to cease intake prior to the study; 4) history of multiple TBI; 5) quadriplegia; 6) body mass index greater than 40 kg/m^2^; 7) pregnancy; 8) recent trans-meridian travel; 9) night or shift work; 10) contraindication to performing an MRI scan; and 11) inability to communicate in French or English. In addition, based on the same eligibility criteria (except for the presence of TBI), a group of 32 healthy controls with similar age and sex distribution was recruited through newspaper advertisements or referrals by participants with TBI. Written consent was obtained from eligible participants, and the study was approved by the research ethics board of the *Centre intégré universitaire de santé et de services sociaux du Nord de l’Île-de-Montréal* [#2011–690] and the *Unité de neuroimagerie fonctionnelle du Centre recherche de l’Institut universitaire de gériatrie de Montréal* [# CMER-RNQ 12–13-001].

### 2.2 Overview of the research design

Participants filled out a sleep diary for seven consecutive days before the study and wore a wrist actigraphy device to measure their sleep–wake patterns. Sleep diary and actigraphy data were used to ensure normal sleep patterns in the study sample and to schedule overnight polysomnography sessions based on participants’ usual bedtime and waketime. These data have been analyzed previously for a larger group of moderate to severe TBI patients and healthy controls (El-Khatib et al., 2019). Participants underwent an MRI scan on the same day as their overnight in-laboratory polysomnography session. The next day, they were tested with a comprehensive neuropsychological assessment and were asked to fill out questionnaires assessing daytime sleepiness, fatigue, and sleep quality, including the Epworth Sleepiness Scale (Johns, 1991), Fatigue Severity Scale (Krupp et al., 1989), and Pittsburgh Sleep Quality Index (Buysse et al., 1989). Participants also filled out the Beck Depression Inventory-II (Beck et al., 1996) and the Beck Anxiety Inventory (Beck et al., 1988) questionnaires.

### 2.3 Data acquisition

#### 2.3.1 Polysomnography

EEG data was acquired from the FP1, FP2, Fz, F3, F4, F7, F8, Cz, C3, C4, Pz, P3, P4, O1, O2, T3, T4, T5, and T6 derivations, linked to mastoid reference electrodes. Commercial software (Harmonie Stellate Systems, Montreal, Canada) was used to digitize signals at a sampling rate of 256 Hz. In addition to the EEG, the overnight polysomnography session included chin and tibia electromyogram, electrooculogram, electrocardiogram, as well as a thoracoabdominal strain gauge, oronasal cannula, and thermistors to measure respiration and a finger pulse oximeter to measure oxygen saturation. Sleep stage and event scoring was performed based on the American Academy of Sleep Medicine scoring manual (Troester et al., 2023), and sleep cycles were detected automatically based on standard criteria (Aeschbach and Borbély, 1993).

#### 2.3.2 MRI

A 3-Tesla MRI scanner (Magnetom Trio, Siemens Healthcare, USA) with a 32-channel head coil was used to acquire a three-dimensional T1-weighted Turbo Flash multi-echo MP-RAGE (magnetization-prepared rapid gradient-echo) sequence using parameters as previously reported by our research group (Baril et al., 2017): voxel size = 1 × 1 × 1 mm; repetition time = 2 530 ms with four echo times of 1.64, 3.50, 5.36, and 7.22 ms; matrix size = 256 × 256; field view = 256 × 256 mm; flip angles = 7 degrees, 176 sagittal orientations; pixel bandwidth = 651 Hz/pixel; total T1 acquisition duration = 373 s.

### 2.4 Data analysis

#### 2.4.1 Sleep spindle detection and spectral analysis

Spindles were detected during NREM sleep (N2 and N3 stages) on artifact-free epochs for the entire night using an automatic detection algorithm (Martin et al., 2013). Specifically, EEG data were band-pass filtered between 11 and 15 Hz using linear phase finite impulse response filters (− 3 dB). The root mean square of the filtered signal was calculated by using a 0.25-second time window. We applied a threshold to the root mean square signal at the 95th percentile and used a minimum duration of 0.5 s to identify spindles. To limit the number of tests, the main analysis was restricted to spindles detected on the central (pooled C3–C4) EEG derivations, where spindle activity is maximal (Troester et al., 2023), though sensitivity analyses were performed on frontal (pooled F3–F4) and parietal (pooled P3–P4) EEG derivations. Spindle density (number/minute of NREM sleep), maximal peak-to-peak amplitude (μV), mean frequency (Hz), and duration (s) were computed. In addition, the Fast Fourier Transform method was used to calculate sigma spectral power (11–16 Hz) on artifact-free epochs during NREM sleep (N2 and N3 stages) for the entire night, averaged across each pair of electrodes. When an electrode in a pair had major artifacts, only one was included in the analyses (central: one TBI and two healthy control participants; frontal: two TBI participants; parietal: one TBI participant). No other data were missing in the present study.

#### 2.4.2 Preprocessing and region-of-interest analysis

The preprocessing of the T1-weighted images and the voxel-wise whole-brain between-group comparison of GMV were performed and described previously for another study with the same participants (Kalantari et al., 2024). Briefly, we used the Computational Anatomy Toolbox (CAT, version 12.7; http://www.neuro.uni-jena.de/cat/) of SPM 12 to preprocess T1-weighted scans involving the following steps: 1) segmentation into gray matter, white matter, and cerebrospinal fluid; 2) spatial registration into a sample-specific template for the entire sample using the DARTEL (Diffeomorphic Anatomical Registration Through Exponentiated Lie Algebra) algorithm (Ashburner, 2007); 3) modulation of normalized gray matter segments; and 4) spatial smoothing using an 8 mm Gaussian filter, performed for voxel-wise whole-brain analysis. The total intracranial volumes (TIV) were estimated using CAT 12 to correct for sex-related differences and variations in brain size.

The first set of regions of interest (ROIs) used in this study consisted of the three clusters with significantly smaller GMV in the TBI group compared to healthy controls (left and right frontotemporal and left temporal), derived from the voxel-wise whole-brain between-group comparison performed in our previous study (Kalantari et al., 2024). These ROIs were chosen due to their significant GMV reduction in the TBI group, thus representing brain areas that were most affected by TBI in our sample. Because of the heterogeneity in brain lesions following moderate to severe TBI, we also studied a second set of ROIs, consisting of anatomical bilateral regions linked with sleep spindles in studies of healthy adults. These anatomical ROIs included the hippocampus, insula, cingulate, supplementary motor area, cerebellum, Heschl’s gyri, thalamus, medial prefrontal cortex (including the medial and medial orbital parts of the frontal superior gyrus), putamen, and pallidum (Fitzroy et al., 2021, Fogel et al., 2017, Muehlroth et al., 2020, Saletin et al., 2013, Toor et al., 2023).

The MARSeille Boîte À Région d'Intérêt (MarsBaR, version 0.45; https://marsbar-toolbox.github.io/index.html) toolbox for SPM 12 was used to create the ROIs. The ROIs derived from the whole-brain analysis were previously defined using the Get SPM Cluster function of the MarsBaR (Kalantari et al., 2024). The bilateral structural ROIs were created from the preprocessed unsmoothed modulated normalized gray matter segments using the Anatomical Automatic Labelling atlas (Tzourio-Mazoyer et al., 2002) implemented in the MarsBaR toolbox. For both sets of ROIs, GMV (in mm^3^) per ROI per subject was extracted using a MATLAB script (Centre for Medical Image Computing, University College London; http://www0.cs.ucl.ac.uk/staff/g.ridgway/vbm/get_totals.m), with a masking threshold to exclude gray matter voxels with signal values below 0.1. For all ROIs, GMV values were divided by TIV per subject to normalize GMV for variations in brain size.

### 2.5 Statistical analysis

IBM SPSS Statistics software (version 29 for Mac; https://www.ibm.com/spss) was used to perform the analyses. The normality of each variable was assessed using the Shapiro-Wilk test (*p* < 0.05). Outliers were identified based on *z*-scores and log transformation was performed to bring extreme values closer to the mean. We performed univariate general linear models controlled for age to compare groups for spindle variables and TIV-normalized GMV in each ROI. When assumptions for the parametric test were violated, Quade nonparametric analysis of covariance (ANCOVA) with age as a covariate was used for between-group comparisons.

We used the PROCESS macro (Hayes, 2022) in SPSS to perform moderation analyses for the relationship between TIV-normalized GMVs and spindle variables with Group as a moderator. For the first set of ROIs, we performed separate moderation analyses (three ROIs x five central spindle variables = 15 models) to test the GMV–spindle associations in regions with GMV loss in the TBI group compared to controls. For the second set of ROIs, we also performed separate moderation analyses (10 ROIs x five central spindle variables = 50 models) to replicate previous findings in studies of healthy adults. Spindle density, characteristics, or sigma power were included in each model as a dependent variable, ROI GMV as an independent variable, Group as a moderator, and age as a covariate. GMV values were mean-centered for GMV X Group interaction analyses. We applied Benjamini-Hochberg’s false discovery rate (FDR) separately per ROI set to adjust for multiple comparisons. We performed post-hoc hierarchical multiple linear regression analyses for significant effects to calculate the percentage of variance in spindle characteristics explained by ROI GMVs and to test the spindle–GMV associations separately per group for significant moderation effects. Age was entered in these models first, followed by GMV. We used two-tailed statistical tests with a significance threshold of *p* < 0.05.

Finally, for sensitivity analysis, we repeated the above analyses for spindle variables calculated from the frontal and parietal EEG derivations. FDR correction was applied separately per ROI set to control for multiple comparisons. As another sensitivity analysis, we performed a Principal Component Analysis (PCA) on the entire sample to account for the potential influence of subjective sleep–wake disturbances and affective symptoms on the GMV–spindle relationships. The PCA included five variables: subjective sleep quality, daytime fatigue, excessive daytime sleepiness, as well as depression and anxiety scores. Sampling adequacy was assessed using the Kaiser-Meyer-Olkin measure, and Bartlett’s test of sphericity was applied to examine correlations among variables. Factors with eigenvalues greater than 1 were retained. The extracted components were then included, along with age, as control variables in moderation models testing the relationships between ROI GMVs and spindle variables with Group as a moderator.

Using PASS software (v23.02), we performed post-hoc sensitivity power analyses to calculate the minimum detectable effect sizes for our main analyses. Assuming a significance level of 5 % with a statistical power of 80 %, the minimum detectable effect sizes were calculated as η^2^_partial_ = 0.12 for between-group comparisons and R^2^_change_ = 0.079 for Group x GMV interaction terms. The minimum detectable effect size for the post-hoc regression analysis for the smaller subgroup (TBI, n = 27) was R^2^_change_ = 0.25.

## 3 Results

### 3.1 Participant characteristics

Participant recruitment has been reported in detail in our previous publication (Kalantari et al., 2024). Twenty-seven participants with chronic moderate to severe TBI were compared to 32 healthy controls of similar age and sex distribution. Participants with TBI had significantly lower education levels compared to healthy controls. They also reported more fatigue, poorer sleep quality, and higher depressive and anxiety symptoms compared to healthy participants. Additionally, polysomnography recording showed more awakenings in participants with TBI compared to healthy controls, but no other between-group differences were observed (Table 1).

**Table 1:** Sample characteristics, questionnaires, and sleep architecture variables.

	Controls	TBI	*t*, x^2^, or *U*	*df*	*p*-values
**Demographics**					
Age, years	29.2 ± 11.5	32.0 ± 12.2	465.0	−	0.62
Sex, women (%)	9 (28.1 %)	8 (29.6 %)	0.02	1	0.90
Education, years	15.3 ± 2.2	12.6 ± 3.4	3.6	43.6	< 0.001*

**Injury variables**					
Time post-injury, months	−	22.6 ± 8.9		−	−
Post-traumatic amnesia, days	−	18.5 ± 17.5	−	−	−
GCS score at hospital admission	−	8.6 ± 3.3	−	−	−

**Questionnaires**					
Fatigue Severity Scale	28.4 ± 10.9	38.1 ± 16.7	− 2.6	43.2	0.01*
Epworth Sleepiness Scale	6.5 ± 4.2	8.3 ± 5.8	497.0	−	0.32
Pittsburgh Sleep Quality Index	4.2 ± 2.0	6.4 ± 3.3	574.0	−	0.01*
Beck Depression Inventory-II	4.2 ± 4.7	16.3 ± 9.7	745.0	−	< 0.001*
Beck Anxiety Inventory	3.6 ± 4.5	10.1 ± 9.7	640.0	−	0.001*

**Sleep architecture**					
Total sleep time, min	432.5 ± 59.8	464.0 ± 64.5	558.0	−	0.06
Wake after sleep onset, min	43.2 ± 33.9	61.2 ± 44.4	551.5	−	0.07
Number of awakenings	25.0 ± 10.0	34.8 ± 13.0	633.5	−	0.002*
Sleep latency, min	25.1 ± 36.3	14.8 ± 15.4	383.0	−	0.46
Sleep efficiency, %	90.7 ± 7.1	88.0 ± 9.2	347.0	−	0.20
N1 sleep, %	9.2 ± 4.1	12.3 ± 6.7	540.0	−	0.10
N2 sleep, %	53.5 ± 7.4	53.3 ± 7.4	0.09	57	0.92
N3 sleep, %	18.1 ± 8.1	15.6 ± 9.9	1.1	57	0.30
REM sleep, %	19.2 ± 5.1	18.8 ± 5.3	0.3	57	0.78
Apnea-hypopnea index, events/h	1.8 ± 2.4	3.0 ± 3.8	476.0	−	0.50

Results are presented as mean ± standard deviation, except for sex, which is presented as number (%). Significant *p* values (< 0.05) are depicted in bold with an asterisk. The degrees of freedom (*df*) are provided for t-tests and the chi-square test. Reproduced from Kalantari et al. (2024).

### 3.2 Group differences in GMV and spindle characteristics

**Fig. 1:**
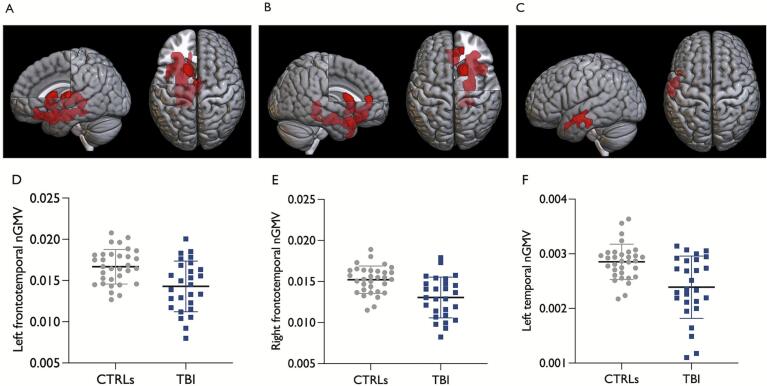
Clusters with significantly smaller GMV in adults with TBI compared to healthy controls. Clusters with significantly smaller GMV in adults with TBI compared to healthy controls (in red in Panels A, B, and C), obtained from voxel-by-voxel whole-brain between-group GMV comparison, were overlayed on the MNI152 T1 template. (A) The Left frontotemporal cluster (K_E_ = 8440, *p*_cluster-level_ < 0.001) included voxels mainly in the nucleus accumbens, caudate, putamen, thalamus, hippocampus, parahippocampus, amygdala, superior temporal pole, medial and posterior orbital gyrus, and rectus in the left hemisphere. (B) The right frontotemporal cluster (K_E_ = 7691, *p*_cluster-level_ < 0.001) included voxels mainly in the superior temporal pole, anterior, medial and posterior orbital gyrus, parahippocampus, hippocampus, amygdala, middle temporal gyrus, anterior cingulate, nucleus accumbens, inferior frontal gyrus pars orbitalis, medial orbital part of superior frontal gyrus, and fusiform in the right hemisphere. (C) The left temporal cluster (K_E_ = 1471, *p*_cluster-level_ < 0.02) included voxels in the left middle and inferior temporal gyrus and the left middle temporal pole. (D-F) Individual values for ROI GMVs, and their means and standard deviations are depicted per group (healthy controls in gray and TBI in blue). GMVs were normalized against the total intracranial volume (GMV in mm^3^/TIV * 1000). CTRLs = controls; TBI = traumatic brain injury; nGMV = normalized gray matter volume; K_E =_ cluster extent threshold. (For interpretation of the references to colour in this figure legend, the reader is referred to the web version of this article.)

A voxel-wise whole-brain comparison of GMV, performed in a previous study of the present sample (Kalantari et al., 2024), revealed three clusters with significantly smaller GMVs in the TBI group compared to healthy controls (Fig. 1). Among the second set of ROIs, GMVs in the hippocampus (*F*_(1, 57)_ = 7.3, *p* = 0.009) and thalamus (*F*_(1, 56)_ = 5.9, *p* = 0.019) were smaller in the TBI group compared to healthy controls, while no between-group differences were found for other ROIs, namely the insula, cingulate, supplementary motor area, cerebellum, Heschl’s gyri, medial prefrontal cortex, putamen, or pallidum (Supp. Table 1). Finally, no significant between-group difference was observed for central spindle characteristics or absolute sigma activity power (Supp. Table 2). Sensitivity analysis for spindle density, characteristics, and sigma power calculated from frontal and parietal EEG derivations revealed similar results, showing no between-group differences (Supp. Table 2).

### 3.3 Relationship between GMV and spindles in control and TBI groups

**Fig. 2:**
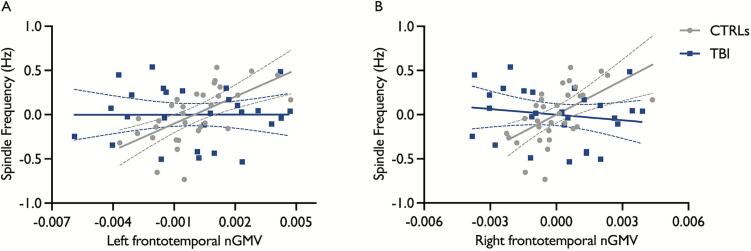
Moderation effect for the relationship between left and right frontotemporal GMVs and central spindle frequency. The residual scatter plots adjusted for age, separately per group, are depicted for the associations between central spindle frequency and (A) left and (B) right frontotemporal clusters for the TBI (blue) and control (gray) groups. GMVs were normalized against the total intracranial volume (GMV in mm^3^/ TIV * 1000). Dotted curves depict 95 % confidence intervals. CTRLs = controls; TBI = traumatic brain injury; nGMV = normalized gray matter volume. (For interpretation of the references to colour in this figure legend, the reader is referred to the web version of this article.)

We performed multiple regression analyses with Group as a moderating factor to test whether the associations between central sleep spindle characteristics and ROI GMVs differed in healthy controls and adults with moderate to severe TBI. For the first set of ROIs, we found that larger left frontotemporal and left temporal GMVs were significantly associated with higher spindle amplitude and sigma power across the entire sample, with no significant Group x GMV interaction. In addition, we found a non-significant trend for the associations between the right frontotemporal cluster and both spindle amplitude and sigma power across the entire sample, with no significant Group x GMV interaction (Table 2, Supp. Fig. 1). However, Group significantly moderated the associations between spindle frequency and left and right frontotemporal GMVs (Fig. 2). Post-hoc regression analyses showed that smaller left and right frontotemporal GMVs were associated with slower spindle frequency in healthy controls (left frontotemporal: *b* = 101.6, *SE* = 24.2, R^2^_change_ = 0.377, *F*_change (1, 29)_ = 17.6, *p* < 0.001; right frontotemporal: *b* = 129.8, *SE* = 35.4, R^2^_change_ = 0.315, *F*_change (1, 29)_ = 13.4, *p* < 0.001) but not in TBI participants (left frontotemporal: *b* = 0.097, *SE* = 21.7, R^2^_change_ = 0.000, *F*_change (1, 24)_ = 0.00, *p* = 1.00; right frontotemporal: *b* = -21.2, *SE* = 27.0, R^2^_change_ = 0.022, *F*_change (1, 24)_ = 0.6, *p* = 0.44). We did not find any significant association between GMV in any of the three clusters and spindle density or duration, nor any significant Group x GMV interactions for these associations.

**Table 2:** Moderation analyses for the relationships between GMVs in ROI set 1 and central spindle characteristics and sigma power.

Regions	*b*	*SE*	*R*^2^_change_	*F*_change (1, 54)_	*p*_FDR-corrected_
**Density, nb/min**					
L. frontotemporal	0.8	20.6	−	−	0.97
L. frontotemporal x Group	34.4	24.5	0.031	2.0	0.38
R. frontotemporal	−13.6	28.0	−	−	0.79
R. frontotemporal x Group	41.4	31.9	0.028	1.7	0.38
L. temporal	37.8	135.2	−	−	0.85
L. temporal x Group	120.4	152.1	0.010	0.6	0.53

**Amplitude, μV**					
L. frontotemporal	2192.6	729.8	−	−	0.012*
L. frontotemporal x Group	−894.0	867.4	0.013	1.1	0.46
R. frontotemporal	2260.9	1009.2	−	−	*0.055*
R. frontotemporal x Group	−860.0	1152.9	0.008	0.6	0.53
L. temporal	16620.5	4698.1	−	−	0.004*
L. temporal x Group	−11783.7	5285.0	0.062	5.0	0.13

**Frequency, Hz**					
L. frontotemporal	105.0	25.8	−	−	0.003*
L. frontotemporal x Group	−106.6	30.6	0.167	12.1	0.008*
R. frontotemporal	125.8	34.5	−	−	0.004*
R. frontotemporal x Group	−145.6	39.4	0.194	13.7	0.008*
L. temporal	355.1	183.7	−	−	0.10
L. temporal x Group	−395.8	206.7	0.062	3.7	0.18

**Duration, s**					
L. frontotemporal	3.4	4.0	−	−	0.54
L. frontotemporal x Group	−3.1	4.7	0.007	0.4	0.56
R. frontotemporal	1.4	5.3	−	−	0.85
R. frontotemporal x Group	−3.2	6.0	0.005	0.3	0.60
L. temporal	24.1	25.6	−	−	0.53
L. temporal x Group	−24.4	28.8	0.012	0.7	0.53

**Sigma power, μV^2^**					
L. frontotemporal	52.2	18.2	−	−	0.015*
L. frontotemporal x Group	−29.6	21.7	0.024	1.9	0.38
R. frontotemporal	56.8	24.8	−	−	*0.055*
R. frontotemporal x Group	−30.8	28.3	0.016	1.2	0.46
L. temporal	362.5	118.0	−	−	0.012*
L. temporal x Group	−289.8	132.8	0.061	4.8	0.13

FDR-corrected statistically significant *p*-values (*p* < 0.05) are depicted in bold with an asterisk. Non-significant FDR-corrected *p* values with a trend towards significance are shown in italics. *b* = unstandardized regression coefficient; *SE* = standard error; R^2^_change_ = change in coefficient of determination; FDR = false discovery rate; L. = left; R. = right.

**Fig. 3:**
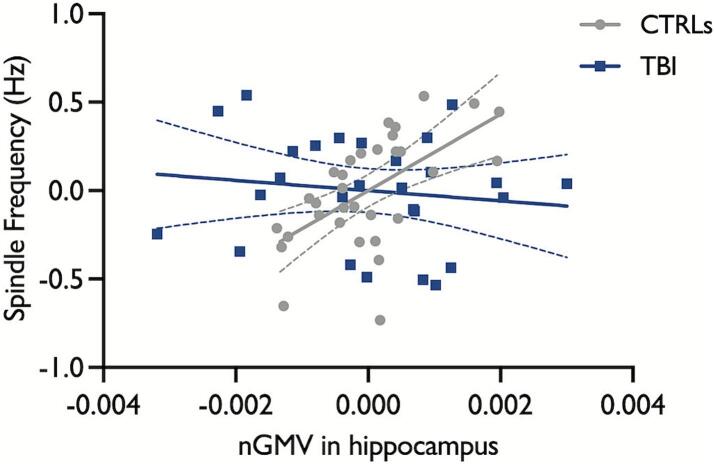
Moderation effect for the relationship between hippocampal GMV and central spindle frequency. The scatter plot depicts residuals adjusted for the effect of age separately per group. Individual data points, regression lines, and 95 % confidence intervals are depicted for TBI (blue) and controls (gray). Hippocampal GMV was normalized against the total intracranial volume (GMV in mm^3^/ TIV * 1000). CTRLs = controls; TBI = traumatic brain injury; nGMV = normalized gray matter volume. (For interpretation of the references to colour in this figure legend, the reader is referred to the web version of this article.)

For the second set of ROIs, we found that larger GMVs in the thalamus and medial prefrontal cortex were associated with higher central spindle amplitude and sigma power across the entire sample (Table 3, Supp. Fig. 2). Larger GMVs in the insula, cingulate, supplementary motor area, Heschl’s gyri, thalamus, medial prefrontal cortex, and putamen were also associated with faster spindle frequency across the entire sample (See Supp. Fig. 2 for examples of these associations). In addition, a significant moderation effect for Group was observed for the association between hippocampal GMV and spindle frequency (Fig. 3). Post-hoc regression analyses demonstrated a positive and significant association between spindle frequency and hippocampal GMV in healthy controls but not in participants with TBI (Controls: *b* = 216.0, *SE* = 54.1, R^2^_change_ = 0.354, *F*_change (1, 29)_ = 15.9, *p* < 0.001; TBI: *b* = -29.0, *SE* = 43.4, R^2^_change_ = 0.016, *F*_change (1, 24)_ = 0.4, *p* = 0.51). No other significant associations or Group x GMV interactions were found.

**Table 3:** Moderation analyses for the relationships between GMVs in ROI set 2 and central spindle characteristics and sigma power.

Regions	*b*	*SE*	*R*^2^_change_	*F*_(1, 54)_	*p*_FDR-corrected_
**Density, nb/min**					
Hippocampus	0.1	46.3	−	−	1.00
Hippocampus x Group	65.9	54.1	0.024	1.5	0.52
Insula	−20.1	24.7	−	−	0.55
Insula x Group	48.7	28.6	0.048	2.9	0.31
Cingulate	−5.8	15.0	−	−	0.78
Cingulate x Group	29.5	17.6	0.046	2.8	0.31
SMA	–23.6	31.4	−	−	0.57
SMA x Group	62.3	39.4	0.042	2.5	0.35
Cerebellum	−4.0	4.7	−	−	0.55
Cerebellum x Group	14.1	5.7	0.093	6.2	0.10
Heschl’s gyri	−116.6	148.8	−	−	0.56
Heschl’s gyri x Group	391.7	191.4	0.067	4.2	0.18
Thalamus	20.9	31.3	−	−	0.62
Thalamus x Group	64.8	42.1	0.036	2.4	0.36
mPFC	−0.2	18.7	−	−	1.00
mPFC x Group	33.9	23.7	0.034	2.0	0.40
Putamen	−42.3	37.7	−	−	0.43
Putamen X Group	102.3	43.6	0.086	5.5	0.11
Pallidum	−255.6	251.8	−	−	0.48
Pallidum X Group	667.6	297.5	0.079	5.0	0.13
**Amplitude, μV**					
Hippocampus	3895.1	1684.9	−	−	0**.***072*
Hippocampus x Group	−1532.9	1971.1	0.008	0.6	0.73
Insula	1549.3	919.3	−	−	0.20
Insula x Group	−395.2	1064.9	0.002	0.1	0.87
Cingulate	1096.7	554.9	−	−	0.12
Cingulate x Group	−161.2	651.5	0.001	0.1	0.87
SMA	1641.6	1184.8	−	−	0.32
SMA x Group	−377.7	1488.1	0.001	0.1	0.87
Cerebellum	156.0	185.1	−	−	0.55
Cerebellum x Group	114.9	223.4	0.004	0.3	0.87
Heschl’s gyri	6577.7	5706.4	−	−	0.43
Heschl’s gyri x Group	1540.9	7342.2	0.001	0.04	0.87
Thalamus	3542.5	1131.1	−	−	0.023*
Thalamus x Group	−1068.5	1522.2	0.006	0.5	0.76
mPFC	1801.6	680.1	−	−	0.044*
mPFC x Group	−687.3	858.7	0.009	0.6	0.73
Putamen	2353.6	1395.2	−	−	0.20
Putamen X Group	447.7	1613.1	0.001	0.08	0.87
Pallidum	18025.7	9177.5	−	−	0.12
Pallidum X Group	2416.1	10844.7	0.001	0.05	0.87

**Frequency, Hz**					
Hippocampus	224.1	57.6	−	−	0.010*
Hippocampus x Group	−256.7	67.4	0.202	14.5	0.020*
Insula	95.7	32.2	−	−	0.028*
Insula x Group	−88.7	37.3	0.088	5.6	0.11
Cingulate	62.3	19.2	−	−	0.020*
Cingulate x Group	−69.8	22.6	0.142	9.6	*0.070*
SMA	121.1	40.7	−	−	0.028*
SMA x Group	−111.0	51.1	0.073	4.7	0.14
Cerebellum	15.1	6.6	−	−	*0.073*
Cerebellum x Group	−15.2	8.0	0.060	3.6	0.22
Heschl’s gyri	637.1	193.3	−	−	0.020*
Heschl’s gyri x Group	−705.6	248.7	0.121	8.0	*0.070*
Thalamus	154.9	41.1	−	−	0.010*
Thalamus x Group	−155.0	55.3	0.113	7.9	*0.070*
mPFC	80.8	24.0	−	−	0.020*
mPFC x Group	−76.7	30.3	0.096	6.4	0.10
Putamen	146.8	50.4	−	−	0.029*
Putamen X Group	−164.9	58.3	0.12	8.0	*0.070*
Pallidum	857.3	339.6	−	−	*0.055*
Pallidum X Group	−1044.0	401.3	0.11	6.8	0.099

**Duration, s**					
Hippocampus	4.3	8.9	−	−	0.73
Hippocampus x Group	−6.2	10.4	0.006	0.4	0.84
Insula	0.9	4.7	−	−	0.91
Insula x Group	−2.9	5.4	0.005	0.3	0.87
Cingulate	−1.3	2.9	−	−	0.74
Cingulate x Group	0.5	3.4	0.0003	0.0	0.91
SMA	0.7	5.9	−	−	0.94
SMA x Group	−2.4	7.5	0.002	0.1	0.87
Cerebellum	−0.8	0.9	−	−	0.55
Cerebellum x Group	1.1	1.1	0.016	0.9	0.70
Heschl’s gyri	−24.3	28.6	−	−	0.55
Heschl’s gyri x Group	26.8	36.8	0.009	0.5	0.76
Thalamus	7.0	6.2	−	−	0.43
Thalamus x Group	−2.8	8.3	0.002	0.1	0.87
mPFC	−0.8	3.6	−	−	0.91
mPFC x Group	−1.1	4.5	0.001	0.1	0.87
Putamen	3.6	7.4	−	−	0.73
Putamen X Group	−3.1	8.5	0.002	0.1	0.87
Pallidum	60.6	48.4	−	−	0.39
Pallidum X Group	−48.3	57.2	0.012	0.7	0.72

**Sigma power, μV^2^**					
Hippocampus	100.6	41.6	−	−	*0.063*
Hippocampus x Group	−62.0	48.6	0.022	1.6	0.49
Insula	39.4	22.7	−	−	0.20
Insula x Group	–23.1	26.3	0.011	0.8	0.72
Cingulate	31.4	13.5	−	−	*0.072*
Cingulate x Group	−13.3	15.8	0.010	0.7	0.72
SMA	56.1	28.5	−	−	0.12
SMA x Group	−37.8	35.8	0.016	1.1	0.65
Cerebellum	7.0	4.5	−	−	0.24
Cerebellum x Group	−2.0	5.4	0.002	0.1	0.87
Heschl’s gyri	149.0	140.6	−	−	0.46
Heschl’s gyri x Group	−15.0	180.9	0.0001	0.0	0.93
Thalamus	79.1	28.5	−	−	0.034*
Thalamus x Group	–33.3	38.3	0.010	0.8	0.72
mPFC	47.0	16.6	−	−	0.032*
mPFC x Group	−31.4	21.0	0.030	2.2	0.37
Putamen	76.0	33.8	−	−	*0.076*
Putamen X Group	−15.7	39.1	0.002	0.16	0.87
Pallidum	554.3	221.2	−	−	*0.055*
Pallidum X Group	−95.0	261.4	0.0017	0.13	0.87

Significant FDR-corrected *p*-values (*p* < 0.05) are depicted in bold with an asterisk. Non-significant FDR-corrected *p* values with a trend towards significance are shown in italics. SMA = supplementary motor area; mPFC = medial prefrontal cortex; *b* = unstandardized regression coefficient; *SE* = standard error; R^2^_change_ = change in coefficient of determination; FDR = false discovery rate; TBI = traumatic brain injury.

Multiple regression analyses also demonstrated that, overall, ROI GMVs explained 9.9 %–17.1 % of the variance in spindle amplitude, 0.9 %–7.7 % of the variance in spindle frequency, and 4.8 %–10.9 % of the variance in sigma power across the entire sample. GMVs in the left and right frontotemporal regions and hippocampus also explained 35.4 %–37.7 % of the variance in spindle frequency in healthy adults only. Furthermore, sensitivity moderation analyses with spindle variables calculated from the frontal and parietal EEG derivations were in line with the main findings for both ROI sets (Supp. Tables 3–6). We observed a greater number of significant effects in the frontal as compared to central and parietal EEG derivations. Particularly, frontal spindle amplitude and sigma power were positively associated with GMV in most ROIs. Associations between ROI GMVs and spindle frequency were also generally similar across EEG derivations, but a few more GMV x Group interactions (i.e., positive associations in healthy controls and absence of associations in TBI) were observed for ROI set 2 for frontal compared to central and parietal EEG derivations. Finally, for sensitivity analysis, we performed a PCA on sleep and mood questionnaire scores to extract components accounting for the greatest variance across these variables. These components were then included as control variables in moderation models testing GMV–spindle relationships. The Kaiser-Meyer-Olkin measure verified sampling adequacy (KMO = 0.72), and Bartlett’s test of sphericity indicated that correlations between items were sufficiently large (X^2^ (10) = 68.5, *p* < 0.001). Two components had eigenvalues greater than 1 and together accounted for 70.1 % of the variance. After varimax rotation, Component 1 was characterized by high loadings on subjective sleep quality, anxiety, and depression, while Component 2 was characterized by high loadings on excessive daytime sleepiness and fatigue. Sensitivity moderation analyses that included these components as control variables, along with age, yielded results consistent with the main analyses (Supp. Tables 7–8).

## 4 Discussion

The TBI group in this study had more awakenings during their polysomnography session and reported poorer sleep quality and more daytime fatigue as compared to control participants. In addition, despite their significant GMV loss, they had very similar sleep spindles to healthy controls. Across the entire sample, larger GMVs in the left frontotemporal and left temporal clusters, as well as in the thalamus and medial prefrontal cortex, were associated with higher spindle amplitude and sigma power, without significant GMV x Group interactions. Similarly, GMVs in the insula, cingulate, supplementary motor area, Heschl’s gyri, thalamus, medial prefrontal cortex, and putamen were associated with faster spindle frequency across the entire sample. However, larger GMVs in the left and right frontotemporal clusters and hippocampus were associated with faster spindle frequency in healthy controls but not TBI participants. Overall, these findings demonstrate positive associations between GMV and spindle amplitude, frequency, and sigma power. However, despite significant gray matter atrophy in the TBI group, these associations were either similar across all participants or absent in the TBI group, indicating that TBI did not result in more pronounced GMV–spindle associations. Along with the absence of between-group differences in sleep spindles, these findings suggest that spindles remain stable despite abnormal gray matter loss.

### 4.1 Sleep spindle characteristics and sigma power were comparable between participants with TBI and healthy controls

We found no significant differences between spindle density, characteristics, or sigma power between TBI and healthy participants, similar to previous findings by our group in a subset of the present sample (Sanchez et al., 2020). These findings suggest that spindles may be resilient to brain alterations following moderate to severe TBI. Nevertheless, it is possible that spindles were affected by brain injury but recovered during the chronic stage of TBI. For example, in a daytime nap study of eight adults with moderate to severe TBI (mean 80 days post-TBI) compared to seven healthy controls, the TBI group had sleep spindles with slower frequency and smaller amplitude (Urakami, 2012). However, at follow-up (mean 151 days post-TBI), no between-group differences in spindles were found (Urakami, 2012). Our group also demonstrated fewer sleep spindles in a sample of 11 hospitalized patients with acute moderate to severe TBI compared to a sample of 43 community-dwelling participants with chronic moderate to severe TBI, although this may also have been a consequence of hospitalization (Sanchez et al., 2022). Further investigations with larger sample sizes are needed to understand the extent to which sleep spindles are resilient to brain atrophy secondary to TBI.

### 4.2 Brain injury had little impact on structural gray matter correlates of sleep spindles

We found that smaller ROI GMVs were associated with lower spindle amplitude and sigma power across the entire sample. Sleep spindles seem to occur primarily locally in restricted cortical regions rather than simultaneously across multiple regions (Nir et al., 2011, Piantoni et al., 2017), with their amplitude positively correlated to the extent of cortical region recruitment (Nir et al., 2011). It is, therefore, possible that smaller GMV results in fewer regions involved in spindle synchronization, leading to lower spindle amplitudes and lower sigma spectral power. We also found that spindle frequency was positively associated with GMV in most ROIs. Spindle frequency depends on the speed at which spindles complete a cycle within the thalamocortical network. Specifically, the thalamic reticular nucleus inhibits thalamocortical neurons, which next triggers their rebound excitatory activity. This rebound activity sends excitatory signals to the thalamic reticular nucleus and corticothalamic neurons, initiating the next spindle cycle (Clawson et al., 2016). Intracellular recordings suggest that spindle frequency depends on the length of hyperpolarization of thalamocortical neurons before their rebound bursts of excitatory activity that sets off a new spindle cycle (Steriade and Llinás, 1988). Slower spindle frequency in adults with smaller GMV may then reflect longer thalamocortical hyperpolarization and a delayed rebound excitatory activity.

Nevertheless, the associations between spindle frequency and GMV in the left and right frontotemporal clusters and hippocampus, regions with significantly lower GMV in TBI compared to controls, were observed only in healthy controls. While a smaller TBI sample size may have contributed to non-significant findings, post-hoc analysis revealed extremely small effect sizes in the TBI group compared to healthy controls for these associations: overall, GMV in these regions explained 0 %–2.2 % of the spindle frequency variance in the TBI group, while it explained 35.4 %–37.7 % of the spindle frequency variance in healthy controls. These effect sizes show that the relationship between GMV in these atrophied regions and spindle frequency is weakened in the TBI group, suggesting that the link between spindle frequency and GMV is not straightforward.

Interestingly, except for the few moderation effects observed for the relationship between spindle frequency and GMV in the left and right frontotemporal regions and hippocampus, the degree of the observed associations was similar between groups, despite significant gray matter atrophy in the TBI group. Furthermore, across the entire sample, GMV explained only a small variance in spindle amplitude (9.9 %–17.1 %), frequency (0.9 %-7.7 %), and sigma power (4.8 %–10.9 %). These findings suggest that the variability in spindle characteristics and sigma power may be better explained by factors other than GMV, such as cerebral white matter structure and genetic contribution to spindle variables and sigma power (Adamczyk et al., 2015, Ambrosius et al., 2008, De Gennaro et al., 2005, De Gennaro et al., 2008, Goldschmied et al., 2021, Purcell et al., 2017, Rusterholz et al., 2018). For example, in a study of the subset of the present sample, more severe thalamocortical white matter damage correlated with shorter spindle duration and slower spindle frequency in the TBI group, the latter possibly suggesting a reduction in conduction speed due to myelin damage (Sanchez et al., 2020). However, these associations were absent in healthy controls (Sanchez et al., 2020), contrary to other studies that demonstrated positive associations between cerebral white matter integrity and sleep spindle density, amplitude, and/or sigma-band power in healthy adults (Gaudreault et al., 2018, Mander et al., 2017, Piantoni et al., 2013, Vien et al., 2019). More importantly, the strong contribution of genetic factors to sleep spindles and sigma power may explain their stability in face of GMV loss due to an external impact following TBI. Sigma activity power (8–16 Hz) is considered an “electroencephalographic fingerprint” of human sleep that maintains trait-like stability from night to night in individuals (De Gennaro et al., 2005, De Gennaro et al., 2008). In addition, the genetic contribution to sleep spindle density, amplitude, duration, and/or frequency has been demonstrated in several studies, although the extent of heritability varies across different spindle characteristics and is dependent on the detector, topography, and spindle type based on their frequency (Adamczyk et al., 2015, Goldschmied et al., 2021, Purcell et al., 2017, Rusterholz et al., 2018).

When comparing our findings with previous studies, we generally did not replicate the associations reported in healthy adults, particularly concerning spindle density and duration. Between-study differences in methodology (for example, small volume cluster versus ROI analysis and differences in spindle detection algorithms), differences in tested EEG electrodes, and the experimental protocol (for example, full-night sleep versus daytime nap) are some of the factors that may have resulted in discrepancies between studies. In addition, four of the five studies quantified spindles during sleep following a memory task (Fitzroy et al., 2021, Fogel et al., 2017, Muehlroth et al., 2020, Toor et al., 2023). Because prior wake learning experiences are proposed to induce changes in sleep spindle expression (see Fernandez and Lüthi (2020) for a review), this difference in experimental protocol may also explain the discrepancies between our findings and previous studies.

Finally, we observed topographical variations in the GMV–spindle associations, such that a greater number of significant effects were observed for frontal EEG derivations compared to central or parietal ones. Topographical variations have also been reported in the studies of GMV and spindle associations in healthy adults (Fitzroy et al., 2021, Fogel et al., 2017). Specifically, a daytime nap study found a greater number of significant associations between GMV and total (11–17 Hz) central spindle frequency than slow (11–14 Hz) frontal or fast (14–17 Hz) parietal spindles (Fogel et al., 2017). Another daytime nap study using high-density EEG found a greater number of associations between GMV and sigma power computed from frontal regions than from lateral centro-posterior regions (Fitzroy et al., 2021). Taken together, these findings underscore the importance of considering topographical variations when assessing the relationship between spindles and brain structure.

### 4.3 Limitations and conclusion

Although the present study investigated the relationship between structural gray matter changes and spindle characteristics, it is important to note that functional brain alterations may also influence spindles. TBI triggers secondary injury mechanisms including neuroinflammation, oxidative stress, ionic and neurotransmitter dysregulation, and excitotoxicity, which contribute to long-term structural and functional brain changes (Ladak et al., 2019, McGuire et al., 2019). Because spindle expression relies on the synchronized activity of inhibitory and excitatory neurons within the cortico-thalamo-cortical network (Clawson et al., 2016), functional alterations impacting neurotransmitters and ionic balance may influence spindle expression. Further investigations are needed to understand how TBI-related functional brain alterations influence spindle dynamics. In addition, chronic brain alterations resulting from these secondary injury mechanisms may have influenced our findings. Specifically, neuroinflammation, which has been documented months to years after TBI (Gentleman et al., 2004, Johnson et al., 2013, Loane et al., 2014, Nagamoto-Combs et al., 2007, Ramlackhansingh et al., 2011), could have affected GMV measures, thereby influencing spindle–GMV associations in the TBI group. However, as these processes were not measured, their impact on our findings remain speculative.

Furthermore, potential confounding variables, including observed group differences in demographic and clinical characteristics (Table 1), may have impacted the present findings. Nevertheless, sensitivity analyses incorporating principal components, derived from sleep and mood questionnaire scores as additional control variables in the moderation models, yielded results consistent with the main analyses. The exclusion of TBI patients who used sleep medication or had MRI contraindications also limits the generalizability of our findings, as these individuals may represent a subgroup with more complex injuries and/or greater comorbidities. Similarly, the generalizability of our findings to women is limited, as most recruited participants were men. In addition, the relatively small sample size in the present study may have reduced our ability to detect weaker moderation effects in the relationship between GMV and spindle variables. A larger sample size would allow stratification by sample characteristics such as medication use, sex, and subjective sleep measures to provide a clearer understanding of their influence on findings and improve generalizability. A larger sample would also facilitate the identification of new brain regions where GMV is associated with spindle density, characteristics, and sigma power through whole-brain voxel-wise analysis.

Overall, in the present study, moderate to severe TBI seemed to have little impact on sleep spindles, sigma power, and their associations with GMV. We observed remarkable similarity in spindle variables between groups. We also observed that the GMV–spindle associations were either similar across the entire sample or absent in the TBI group, despite their significant GMV loss. Together, our findings suggest that spindles are stable against significant gray matter damage secondary to moderate to severe TBI. They also suggest that factors other than GMV may better explain spindle characteristics and sigma power. Future experimental research is needed to replicate these findings and to better understand the connection between spindles and brain structure in the context of moderate to severe TBI.

## Funding sources

The authors disclosed receipt of the following financial support for the research, authorship, and/or publication of this article: This work was supported by the Canadian Institutes of Health Research grants [# FDN154291 and # MOP115172], Canada Research Chair in Sleep Disorders and Brain Health, and Fonds de recherche du Québec – santé [# 34851] to NG as a principal investigator; studentships from the Canadian Institutes of Health Research, Quebec Bio-Imaging Network recruitment scholarship, and the J. A. De Sève bursary (Centre Intégré Universitaire de Santé et de Services Sociaux du Nord de l’Île-de Montréal) to NK; postdoctoral fellowships from the Fonds de Recherche du Québec – Santé, Fonds de Recherche du Québec – Nature et Technologies through the Merit scholarship program for foreign students, and Quebec Bio-Imaging network to Claire A; Studentships and postdoctoral fellowship from the Canadian Institutes of Health Research and studentships from the Fonds de recherche du Québec – santé to ES; postdoctoral fellowship from the Canadian Institutes of Health Research to Caroline A.

## CRediT authorship contribution statement

**Narges Kalantari:** Writing – review & editing, Writing – original draft, Methodology, Formal analysis, Conceptualization. **Véronique Daneault:** Writing – review & editing, Methodology. **Hélène Blais:** Writing – review & editing, Formal analysis. **Claire André:** Writing – review & editing, Methodology. **Erlan Sanchez:** Writing – review & editing, Investigation. **Jean-Marc Lina:** Writing – review & editing, Methodology. **Caroline Arbour:** Writing – review & editing, Investigation. **Danielle Gilbert:** Writing – review & editing, Investigation. **Julie Carrier:** Writing – review & editing, Supervision, Conceptualization. **Nadia Gosselin:** Writing – review & editing, Supervision, Project administration, Methodology, Funding acquisition, Conceptualization.

## Declaration of competing interest

The authors declare the following financial interests/personal relationships which may be considered as potential competing interests: Nadia Gosselin reports funding grants from Eisai, Idorsia, the Weston Family Foundation, and the American Academy of Sleep Medicine Foundation. Nadia Gosselin has received honoraria from Eisai and sponsorships from Jazz Pharmaceuticals, Eisai, Axsome, Idorsia, and Paladin. Claire Andre reports board membership with the Alzheimer’s Association (ISTAART Group, Sleep and Circadian Rhythms). None of these contributions are related to the present study. All other authors declare no known competing financial interests or personal relationships that could have influenced the work reported in this paper.

## Data Availability

Data will be made available on request.

## References

[b0005] Adamczyk M., Genzel L., Dresler M., Steiger A., Friess E. (2015). Automatic sleep spindle detection and genetic influence estimation using continuous wavelet transform. Front. Hum. Neurosci..

[b0010] Aeschbach D., Borbély A.A. (1993). All-night dynamics of the human sleep EEG. J. Sleep Res..

[b0015] Ambrosius U., Lietzenmaier S., Wehrle R., Wichniak A., Kalus S., Winkelmann J., Bettecken T., Holsboer F., Yassouridis A., Friess E. (2008). Heritability of sleep electroencephalogram. Biol. Psychiatry.

[b0020] Ashburner J. (2007). A fast diffeomorphic image registration algorithm. Neuroimage.

[b0025] Baril A.A., Gagnon K., Brayet P., Montplaisir J., De Beaumont L., Carrier J., Lafond C., L'Heureux F., Gagnon J.F., Gosselin N. (2017). Gray matter hypertrophy and thickening with obstructive sleep apnea in middle-aged and older adults. Supplement. Am. J. Respir. Crit. Care Med..

[b0030] Beck A.T., Epstein N., Brown G., Steer R.A. (1988). An inventory for measuring clinical anxiety: psychometric properties. J. Consult. Clin. Psychol..

[b0035] Beck A.T., Steer R.A., Ball R., Ranieri W.F. (1996). Comparison of beck depression inventories-IA and-II in psychiatric outpatients. J. Pers. Assess..

[b0040] Buysse D.J., Reynolds C.F., Monk T.H., Berman S.R., Kupfer D.J. (1989). The Pittsburgh sleep quality index: a new instrument for psychiatric practice and research. Psychiatry Res..

[b0045] Carrier J., Land S., Buysse D.J., Kupfer D.J., Monk T.H. (2001). The effects of age and gender on sleep EEG power spectral density in the middle years of life (ages 20-60 years old). Psychophysiology.

[b0050] Clawson B.C., Durkin J., Aton S.J. (2016). Form and function of sleep spindles across the lifespan. Neural Plast..

[b0055] Crowley K., Trinder J., Kim Y., Carrington M., Colrain I.M. (2002). The effects of normal aging on sleep spindle and K-complex production. Clin. Neurophysiol..

[b0060] Dang-Vu T.T., McKinney S.M., Buxton O.M., Solet J.M., Ellenbogen J.M. (2010). Spontaneous brain rhythms predict sleep stability in the face of noise. Curr. Biol..

[b0065] De Gennaro L., Ferrara M., Vecchio F., Curcio G., Bertini M. (2005). An electroencephalographic fingerprint of human sleep. Neuroimage.

[b0070] De Gennaro L., Marzano C., Fratello F., Moroni F., Pellicciari M.C., Ferlazzo F., Costa S., Couyoumdjian A., Curcio G., Sforza E., Malafosse A., Finelli L.A., Pasqualetti P., Ferrara M., Bertini M., Rossini P.M. (2008). The electroencephalographic fingerprint of sleep is genetically determined: a twin study. Ann. Neurol..

[b0075] El-Khatib H., Arbour C., Sanchez E., Dumont M., Duclos C., Blais H., Carrier J., Paquet J., Gosselin N. (2019). Towards a better understanding of increased sleep duration in the chronic phase of moderate to severe traumatic brain injury: an actigraphy study. Sleep Med..

[b0080] Fernandez L.M.J., Lüthi A. (2020). Sleep spindles: mechanisms and functions. Physiol. Rev..

[b0085] Fitzroy A.B., Kainec K.A., Spencer R.M.C. (2021). Ageing-related changes in nap neuroscillatory activity are mediated and moderated by grey matter volume. Eur. J. Neurosci..

[b0090] Fogel S.M., Vien C., Karni A., Benali H., Carrier J., Doyon J. (2017). Sleep spindles: a physiological marker of age-related changes in gray matter in brain regions supporting motor skill memory consolidation. Neurobiol. Aging.

[b0095] Gaudreault P.O., Gosselin N., Lafortune M., Deslauriers-Gauthier S., Martin N., Bouchard M., Dubé J., Lina J.M., Doyon J., Carrier J. (2018). The association between white matter and sleep spindles differs in young and older individuals. Sleep.

[b0100] Gentleman S.M., Leclercq P.D., Moyes L., Graham D.I., Smith C., Griffin W.S., Nicoll J.A. (2004). Long-term intracerebral inflammatory response after traumatic brain injury. Forensic Sci. Int..

[b0105] Goldschmied J.R., Lacourse K., Maislin G., Delfrate J., Gehrman P., Pack F.M., Staley B., Pack A.I., Younes M., Kuna S.T., Warby S.C. (2021). Spindles are highly heritable as identified by different spindle detectors. Sleep.

[b0110] Guazzelli M., Feinberg I., Aminoff M., Fein G., Floyd T.C., Maggini C. (1986). Sleep spindles in normal elderly: comparison with young adult patterns and relation to nocturnal awakening, cognitive function and brain atrophy. Electroencephalogr. Clin. Neurophysiol..

[b0115] Harris T.C., de Rooij R., Kuhl E. (2019). The shrinking brain: cerebral atrophy following traumatic brain injury. Ann. Biomed. Eng..

[b0120] Hayes, A.F., 2022. Introduction to Mediation, Moderation, and Conditional Process Analysis (T. D. Little, Ed. 3 ed.). Guilford Press.

[b0125] Johns M.W. (1991). A new method for measuring daytime sleepiness: the Epworth sleepiness scale. Sleep.

[b0130] Johnson V.E., Stewart J.E., Begbie F.D., Trojanowski J.Q., Smith D.H., Stewart W. (2013). Inflammation and white matter degeneration persist for years after a single traumatic brain injury. Brain.

[b0135] Kalantari N., Daneault V., Blais H., André C., Sanchez E., Lina J.-M., Arbour C., Gilbert D., Carrier J., Gosselin N. (2024). Cerebral grey matter may not explain sleep slow-wave characteristics after severe brain injury. J. Neurosci..

[b0140] Krupp L.B., LaRocca N.G., Muir-Nash J., Steinberg A.D. (1989). The fatigue severity scale: application to patients with multiple sclerosis and systemic lupus erythematosus. Arch. Neurol..

[b0145] Ladak A.A., Enam S.A., Ibrahim M.T. (2019). A review of the molecular mechanisms of traumatic brain injury. World Neurosurg..

[b0150] Loane D.J., Kumar A., Stoica B.A., Cabatbat R., Faden A.I. (2014). Progressive neurodegeneration after experimental brain trauma: association with chronic microglial activation. J. Neuropathol. Exp. Neurol..

[b0155] Mander B.A., Zhu A.H., Lindquist J.R., Villeneuve S., Rao V., Lu B., Saletin J.M., Ancoli-Israel S., Jagust W.J., Walker M.P. (2017). White matter structure in older adults moderates the benefit of sleep spindles on motor memory consolidation. J. Neurosci..

[b0160] Martin N., Lafortune M., Godbout J., Barakat M., Robillard R., Poirier G., Bastien C., Carrier J. (2013). Topography of age-related changes in sleep spindles. Neurobiol. Aging.

[b0165] McGuire J.L., Ngwenya L.B., McCullumsmith R.E. (2019). Neurotransmitter changes after traumatic brain injury: an update for new treatment strategies. Mol. Psychiatry.

[b0170] Muehlroth B.E., Sander M.C., Fandakova Y., Grandy T.H., Rasch B., Lee Shing Y., Werkle-Bergner M. (2020). Memory quality modulates the effect of aging on memory consolidation during sleep: reduced maintenance but intact gain. Neuroimage.

[b0175] Nagamoto-Combs K., McNeal D.W., Morecraft R.J., Combs C.K. (2007). Prolonged microgliosis in the rhesus monkey central nervous system after traumatic brain injury. J. Neurotrauma.

[b0180] Academies N. (2019). Evaluation of the disability determination process for traumatic brain injury in veterans. The National Academies Press.

[b0185] Nicolas A., Petit D., Rompré S., Montplaisir J. (2001). Sleep spindle characteristics in healthy subjects of different age groups. Clin. Neurophysiol..

[b0190] Nir Y., Staba R.J., Andrillon T., Vyazovskiy V.V., Cirelli C., Fried I., Tononi G. (2011). Regional slow waves and spindles in human sleep. Neuron.

[b0195] Piantoni G., Halgren E., Cash S.S. (2017). Spatiotemporal characteristics of sleep spindles depend on cortical location. Neuroimage.

[b0200] Piantoni G., Poil S.S., Linkenkaer-Hansen K., Verweij I.M., Ramautar J.R., Van Someren E.J., Van Der Werf Y.D. (2013). Individual differences in white matter diffusion affect sleep oscillations. J. Neurosci..

[b0205] Principe J.C., Smith J.R. (1982). Sleep spindle characteristics as a function of age. Sleep.

[b0210] Purcell S.M., Manoach D.S., Demanuele C., Cade B.E., Mariani S., Cox R., Panagiotaropoulou G., Saxena R., Pan J.Q., Smoller J.W., Redline S., Stickgold R. (2017). Characterizing sleep spindles in 11,630 individuals from the National Sleep Research Resource. Nat. Commun..

[b0215] Rabinowitz A.R., Levin H.S. (2014). Cognitive sequelae of traumatic brain injury. Psychiatr. Clin. North Am..

[b0220] Ramlackhansingh A.F., Brooks D.J., Greenwood R.J., Bose S.K., Turkheimer F.E., Kinnunen K.M., Gentleman S., Heckemann R.A., Gunanayagam K., Gelosa G., Sharp D.J. (2011). Inflammation after trauma: microglial activation and traumatic brain injury. Ann. Neurol..

[b0225] Rusterholz T., Hamann C., Markovic A., Schmidt S.J., Achermann P., Tarokh L. (2018). Nature and nurture: brain region-specific inheritance of sleep neurophysiology in adolescence. J. Neurosci..

[b0230] Saletin J.M., van der Helm E., Walker M.P. (2013). Structural brain correlates of human sleep oscillations. Neuroimage.

[b0235] Sanchez E., Arbour C., El-Khatib H., Marcotte K., Blais H., Baril A.A., Bedetti C., Descoteaux M., Lina J.M., Gilbert D., Carrier J., Gosselin N. (2020). Sleep spindles are resilient to extensive white matter deterioration. Brain Commun..

[b0240] Sanchez E., Blais H., Duclos C., Arbour C., Van Der Maren S., El-Khatib H., Baril A.A., Bernard F., Carrier J., Gosselin N. (2022). Sleep from acute to chronic traumatic brain injury and cognitive outcomes. Sleep.

[b0245] Sanchez E., El-Khatib H., Arbour C., Bedetti C., Blais H., Marcotte K., Baril A., Descoteaux M., Gilbert D., Carrier J., Gosselin N. (2019). Brain white matter damage and its association with neuronal synchrony during sleep. Brain.

[b0250] Sandsmark D.K., Elliott J.E., Lim M.M. (2017). Sleep-wake disturbances after traumatic brain injury: synthesis of human and animal studies. Sleep.

[b0255] Steriade M., Llinás R.R. (1988). The functional states of the thalamus and the associated neuronal interplay. Physiol. Rev..

[b0260] Toor B., van den Berg N., Ray L.B., Fogel S.M. (2023). Sleep spindles and slow waves are physiological markers for age-related changes in gray matter in brain regions supporting problem-solving skills. Learn. Mem..

[b0265] Troester M.M., Quan S.F., Berry R.B., Plante D.T., Abreu A.R., Alzoubaidi M., Bandyopadhyay A., DelRosso L., Ebben M., Kwon Y., Mao M., Munir S.S., Pressman M.R., Rodriguez A.J., Ryals S., So J.Y., Vaughn B.V., Thomas S.M. (2023). The AASM Manual for the scoring of sleep and associated events, version 3. American Academy of Sleep Medicine.

[b0270] Tzourio-Mazoyer N., Landeau B., Papathanassiou D., Crivello F., Etard O., Delcroix N., Mazoyer B., Joliot M. (2002). Automated anatomical labeling of activations in SPM using a macroscopic anatomical parcellation of the MNI MRI single-subject brain. Neuroimage.

[b0275] Urakami Y. (2012). Relationship between sleep spindles and clinical recovery in patients with traumatic brain injury: a simultaneous EEG and MEG study. Clin. EEG Neurosci..

[b0280] Vien C., Boré A., Boutin A., Pinsard B., Carrier J., Doyon J., Fogel S. (2019). Thalamo-cortical white matter underlies motor memory consolidation via modulation of sleep spindles in young and older adults. Neuroscience.

[b0285] Wei H.G., Riel E., Czeisler C.A., Dijk D.-J. (1999). Attenuated amplitude of circadian and sleep-dependent modulation of electroencephalographic sleep spindle characteristics in elderly human subjects. Neurosci. Lett..

