# Supplementary material for: Sleep spindle density and morphology are resilient to post-traumatic gray matter volume loss
Source: Neuroimage Clin. 2025 Nov 26;48:103915. doi: 10.1016/j.nicl.2025.103915 (PMC12718450; doi:10.1016/j.nicl.2025.103915)
Supplement: Supplementary Data 1 [file mmc1.docx]

# Supplementary Material

**Supplementary Table 1** Between-group comparison of GMVs for ROI set 2, derived from previous studies of the GMV–spindle associations in healthy adults

| ROI | Controls | TBI |  |  |  |  |
| --- | --- | --- | --- | --- | --- | --- |
|  | Mean ± SD | Mean ± SD | *F-*value | *df* | *p*-value | η^2^ _partial_ |
| Hippocampus | 0.0081 ± 0.00093 | 0.0072 ± 0.0015 | 7.3 | 1, 57 | **.009*** | .10 |
| Insula | 0.017 ± 0.0018 | 0.016 ± 0.0027 | 1.1 | 1, 57 | .31 | .011 |
| Cingulate | 0.028 ± 0.0031 | 0.026 ± 0.0040 | 2.2 | 1, 57 | .15 | .038 |
| SMA | 0.011 ± 0.0014 | 0.011 ± 0.0017 | 0.1 | 1, 56 | .76 | .002 |
| Cerebellum | 0.074 ± 0.0089 | 0.070 ± 0.013 | 1.3 | 1, 56 | .26 | .023 |
| Heschl’s gyri | 0.0023 ± 0.00029 | 0.0022 ± 0.00035 | 0.8 | 1, 56 | .36 | .015 |
| Thalamus | 0.0069 ± 0.0013 | 0.0059 ± 0.0015 | 5.9 | 1, 56 | **.019*** | .095 |
| mPFC | 0.020 ± 0.0024 | 0.019 ± 0.0028 | 0.6 | 1, 56 | .45 | .010 |
| Putamen | 0.0083 ± 0.0012 | 0.0076 ± 0.0017 | 2.5 | 1, 57 | .12 | .042 |
| Pallidum | 0.0012 ± 0.00018 | 0.0011± 0.00024 | 2.8 | 1, 57 | .097 | .039 |

GMV (in mm3) is normalized against the total intracranial volume (GMV * 1000 /TIV). For GMVs of the hippocampus, insula, cingulate, putamen, and pallidum, Levene’s test was significant (*p* < .05), indicating that the homogeneity of variance assumption for ANCOVA was violated. Therefore, nonparametric Quade ANCOVA was performed to compare groups for these variables. Significant *p* values (< .05) are shown in bold with an asterisk. SMA = supplementary motor area; mPFC = medial prefrontal cortex; TBI = traumatic brain injury.

**Supplementary Table 2** Between-group comparison of sleep spindles and sigma power

|  | Controls | TBI |  |  |  |  |
| --- | --- | --- | --- | --- | --- | --- |
| Spindle characteristics | Mean ± SD | Mean ± SD | *F-*value | *df* | *p*-value | η^2^ _partial_ |
| **Main analysis: Central** | | | | | | |
| Density, nb/min | 3.15 ± 0.22 | 3.11 ± 0.26 | 0.2 | 1, 56 | .63 | .004 |
| Amplitude, μV | 33.36 ± 9.34 | 32.15 ± 9.67 | 0.03 | 1, 56 | .88 | .000 |
| Frequency, Hz | 13.08 ± 0.31 | 13.11 ± 0.33 | 0.06 | 1, 56 | .80 | .001 |
| Duration, s | 0.83 ± 0.050 | 0.84 ± 0.037 | 0.9 | 1, 57 | .34 | .004 |
| Sigma power, μV^2^ | 16.29 ± 8.07 | 16.33 ± 9.11 | 0.04 | 1, 56 | .84 | .001 |
| **Sensitivity analysis: Frontal** | | | | | | |
| Density, nb/min | 3.26 ± 0.23 | 3.12 ± 0.33 | 1.6 | 1, 57 | .21 | .043 |
| Amplitude, μV | 37.85 ± 10.5 | 33.83 ± 11.06 | 1.2 | 1, 56 | .27 | .022 |
| Frequency, Hz | 12.66 ± 0.30 | 12.67 ± 0.26 | 0.008 | 1, 56 | .93 | .000 |
| Duration, s | 0.84 ± 0.04 | 0.84 ± 0.05 | 0.08 | 1, 56 | .78 | .001 |
| Sigma power, μV^2^ | 18.97 ± 8.97 | 17.34 ± 9.94 | 0.06 | 1, 56 | .81 | .001 |
| **Sensitivity analysis: Parietal** | | | | | | |
| Density, nb/min | 3.19 ± 0.18 | 3.19 ± 0.20 | 0.01 | 1, 56 | .92 | .000 |
| Amplitude, μV | 32.54 ± 9.75 | 32.31 ± 9.86 | 0.02 | 1, 56 | .88 | .000 |
| Frequency, Hz | 13.32 ± 0.41 | 13.30 ± 0.44 | 0.00 | 1, 57 | .99 | .002 |
| Duration, s | 0.87 ± 0.06 | 0.87 ± .04 | 1.0 | 1, 57 | .33 | .002 |
| Sigma power, μV^2^ | 16.10 ± 8.87 | 16.38 ± 9.8 | 0.1 | 1, 56 | .73 | .002 |

For non-normally distributed variables, including central spindle duration, frontal spindle density, and parietal spindle frequency and duration, the Quade nonparametric analysis of covariance (ANCOVA) was used. The central and parietal sigma spectral power were log-transformed to bring extreme values closer to the mean. The mean ± SD values reported here refer to the non-transformed variables. TBI = traumatic brain injury.

**Supplementary Figure 1** Relationship between GMV in ROI set 1 and central spindle amplitude and sigma power across the entire sample


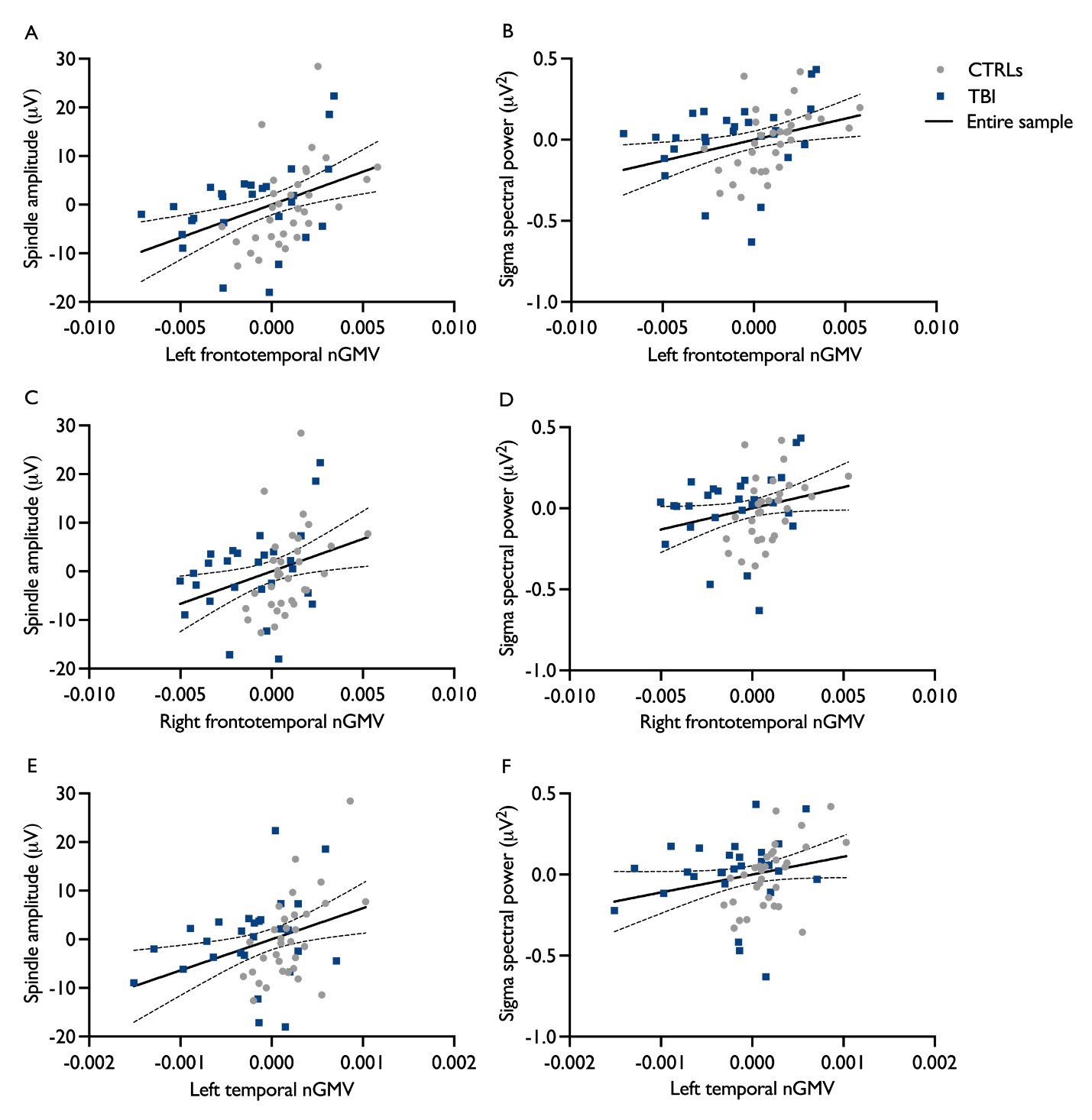


Scatter plots depict residuals adjusted for the effect of age for the entire sample (TBI in blue and controls in grey). GMVs were normalized against the total intracranial volume (GMV in mm^3^/ TIV * 1000). Dotted curves depict 95% confidence intervals. (A & B) Larger left frontotemporal and (E & F) left temporal clusters were significantly associated with higher spindle amplitude and sigma power. (C & D) A larger right frontotemporal cluster was marginally associated (nonsignificant trend) with larger spindle amplitude and sigma power. CTRLs = controls; TBI = traumatic brain injury; nGMV = normalized grey matter volume.

**Supplementary Figure 2** Relationship between GMV in ROI set 2 and central sleep spindle characteristics and sigma power across the entire sample


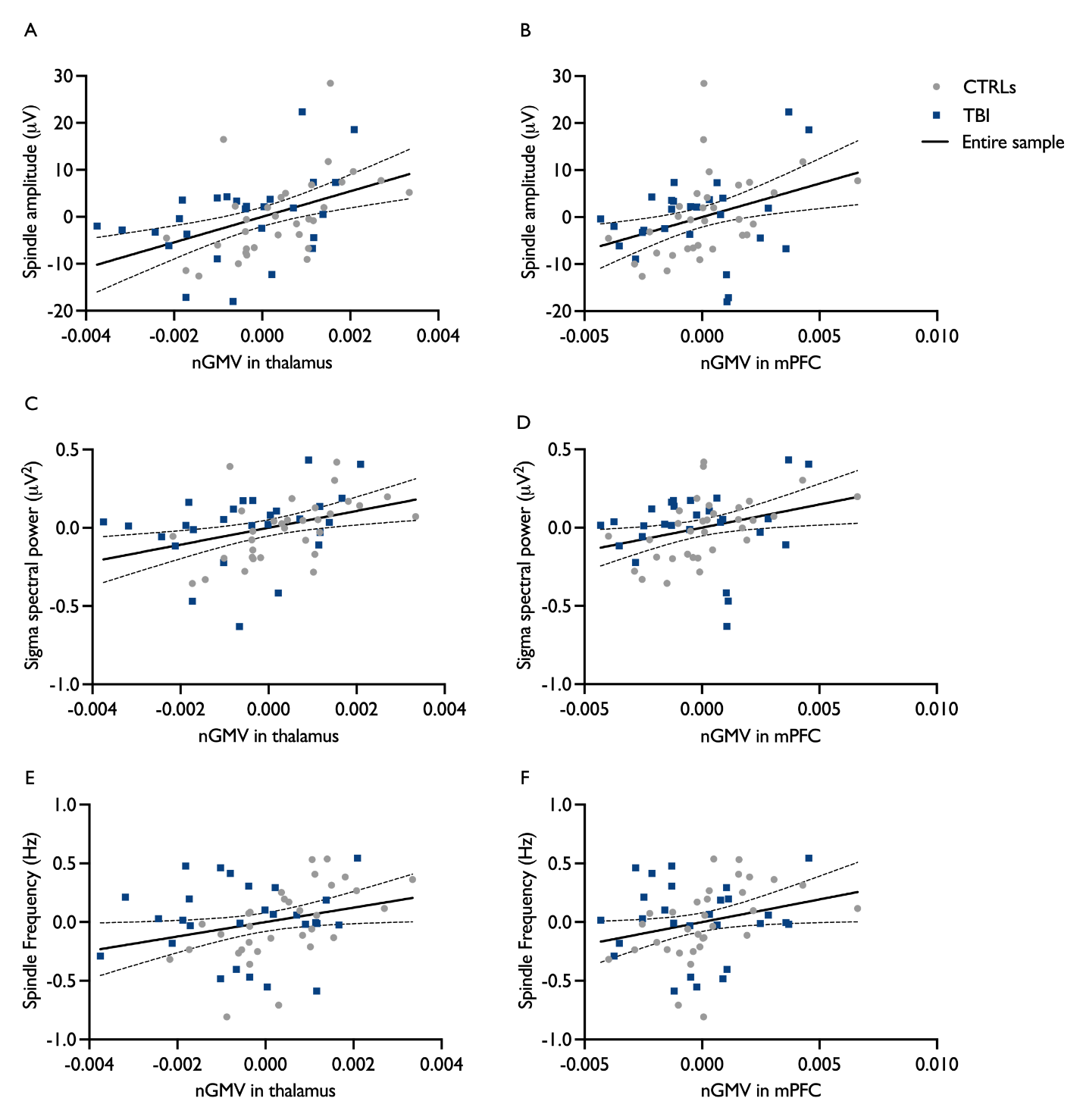


The scatter plots depict residuals adjusted for the effect of age for the entire group (TBI in blue and controls in grey). GMVs were normalized against the total intracranial volume (GMV in mm^3^/ TIV * 1000). Dotted curves depict 95% confidence intervals. (A & B) Associations between spindle amplitude and GMV; (C & D) Associations between sigma power and GMV. (E & F) Examples of the associations between GMV and spindle frequency. CTRLs = controls; TBI = traumatic brain injury; nGMV = normalized grey matter volume; mPFC = medial prefrontal cortex.

**Supplementary Table 3** Moderation analyses for the relationships between GMVs in ROI set 1 and frontal spindle characteristics and sigma power

| Regions | *b* | *SE* | *R*^2^ _change_ | *F* _change (1, 54)_ | *p* _FDR-corrected_ |
| --- | --- | --- | --- | --- | --- |
| **Density, nb/min** | | | | | |
| L. frontotemporal | 25.5 | 23.6 | - | - | .41 |
| L. frontotemporal x Group | 24.3 | 28.0 | .011 | 0.7 | .56 |
| R. frontotemporal | 22.3 | 32.4 | - | - | .57 |
| R. frontotemporal x Group | 24.1 | 37.0 | .006 | 0.4 | .60 |
| L. temporal | 149.5 | 155.2 | - | - | .42 |
| L. temporal x Group | 89.1 | 174.6 | .004 | 0.3 | .63 |
| **Amplitude, μV** | | | | | |
| L. frontotemporal | 3004.0 | 732.1 | - | - | **.001*** |
| L. frontotemporal x Group | -1443.5 | 870.2 | .026 | 2.8 | .24 |
| R. frontotemporal | 3655.7 | 1005.8 | - | - | **.002*** |
| R. frontotemporal x Group | -1851.1 | 1148.9 | .027 | 2.6 | .24 |
| L. temporal | 20813.8 | 4745.0 | - | - | **.001*** |
| L. temporal x Group | -13927.5 | 5337.8 | .065 | 6.8 | *.059* |
| **Frequency, Hz** | | | | | |
| L. frontotemporal | 103.3 | 22.0 | - | - | **< .001*** |
| L. frontotemporal x Group | -100.2 | 26.2 | .193 | 14.6 | **.006*** |
| R. frontotemporal | 122.2 | 30.1 | - | - | **.001*** |
| R. frontotemporal x Group | -131.0 | 34.3 | .204 | 14.6 | **.006*** |
| L. temporal | 388.1 | 159.7 | - | - | **.037*** |
| L. temporal x Group | -428.4 | 179.7 | .094 | 5.7 | *.078* |
| **Duration, s** | | | | | |
| L. frontotemporal | -3.1 | 3.9 | - | - | .53 |
| L. frontotemporal x Group | 4.9 | 4.7 | .018 | 1.1 | .45 |
| R. frontotemporal | -3.4 | 5.2 | - | - | .57 |
| R. frontotemporal x Group | 4.2 | 6.0 | .008 | 0.5 | .59 |
| L. temporal | 5.6 | 25.5 | - | - | .83 |
| L. temporal x Group | 4.8 | 28.7 | .001 | 0.03 | .87 |
| **Sigma power, μV^2^** |  |  |  |  |  |
| L. frontotemporal | 2305.2 | 665.9 | - | - | **.004*** |
| L. frontotemporal x Group | -1028.6 | 791.5 | .018 | 1.7 | .35 |
| R. frontotemporal | 2775.2 | 904.3 | - | - | **.008*** |
| R. frontotemporal x Group | -1239.2 | 1033.1 | .016 | 1.4 | .39 |
| L. temporal | 17960.5 | 4204.4 | - | - | **.001*** |
| L. temporal x Group | -12764.7 | 4729.7 | .074 | 7.3 | *.056* |

FDR-corrected statistically significant *p*-values (*p* < .05) are depicted in bold with an asterisk. Nonsignificant FDR-corrected *p* values with a trend towards significance are shown in italics. *b* = unstandardized regression coefficient; *SE* = standard error; R^2^_change_ = change in coefficient of determination; FDR = false discovery rate; L. = left; R. = right.

**Supplementary Table 4** Moderation analyses for the relationships between GMVs in ROI set 1 and parietal spindle characteristics and sigma power

| Regions | *b* | *SE* | *R*^2^ _change_ | *F* _change (1, 54)_ | *p* _FDR-corrected_ |
| --- | --- | --- | --- | --- | --- |
| **Density, nb/min** | | | | | |
| L. frontotemporal | -16.8 | 17.4 | - | - | .42 |
| L. frontotemporal x Group | 37.6 | 20.7 | .057 | 3.3 | .20 |
| R. frontotemporal | -34.4 | 23.2 | - | - | .23 |
| R. frontotemporal x Group | 44.8 | 26.5 | .050 | 2.9 | .24 |
| L. temporal | -45.7 | 112.6 | - | - | .71 |
| L. temporal x Group | 172.2 | 126.6 | .032 | 1.8 | .34 |
| **Amplitude, μV** | | | | | |
| L. frontotemporal | 2040.7 | 803.6 | - | - | **.030*** |
| L. frontotemporal x Group | -736.0 | 955.1 | .009 | 0.6 | .57 |
| R. frontotemporal | 2006.5 | 1106.4 | - | - | .13 |
| R. frontotemporal x Group | -695.2 | 1263.9 | .005 | 0.3 | .63 |
| L. temporal | 17099.3 | 5120.9 | - | - | **.005*** |
| L. temporal x Group | -12733.5 | 5760.6 | .068 | 4.9 | .10 |
| **Frequency, Hz** | | | | | |
| L. frontotemporal | 127.0 | 34.4 | - | - | **.002*** |
| L. frontotemporal x Group | -126.5 | 40.8 | .134 | 9.6 | **.023*** |
| R. frontotemporal | 168.9 | 44.9 | - | - | **.002*** |
| R. frontotemporal x Group | -187.0 | 51.3 | .182 | 13.3 | **.006*** |
| L. temporal | 492.8 | 239.1 | - | - | *.078* |
| L. temporal x Group | -500.3 | 269.0 | .056 | 3.5 | .20 |
| **Duration, s** | | | | | |
| L. frontotemporal | 6.6 | 4.8 | - | - | .26 |
| L. frontotemporal x Group | -4.7 | 5.7 | 0.011 | 0.7 | .56 |
| R. frontotemporal | 3.9 | 6.4 | - | - | .58 |
| R. frontotemporal x Group | -3.7 | 7.3 | .004 | 0.3 | .63 |
| L. temporal | 31.5 | 31.1 | - | - | .42 |
| L. temporal x Group | -26.4 | 35.0 | .010 | 0.6 | .57 |
| **Sigma power, μV^2^** |  |  |  |  |  |
| L. frontotemporal | 55.9 | 19.7 | - | - | **.015*** |
| L. frontotemporal x Group | -33.1 | 23.4 | .028 | 2.0 | .33 |
| R. frontotemporal | 58.8 | 26.9 | - | - | *.063* |
| R. frontotemporal x Group | -36.0 | 30.8 | .020 | 1.4 | .39 |
| L. temporal | 404.6 | 126.8 | - | - | **.007*** |
| L. temporal x Group | -352.8 | 142.6 | .084 | 6.1 | *.071* |

FDR-corrected statistically significant *p*-values (*p* < .05) are depicted in bold with an asterisk. Non-significant FDR-corrected *p* values with a trend towards significance are shown in italics. *b* = unstandardized regression coefficient; *SE* = standard error; R^2^_change_ = change in coefficient of determination; FDR = false discovery rate; L. = left; R. = right.

**Supplementary Table 5** Moderation analyses for the relationships between GMVs in ROI set 2 and frontal spindle characteristics and sigma power

| Regions | *b* | *SE* | *R*^2^ _change_ | *F* _(1, 54)_ | *p* _FDR-corrected_ |
| --- | --- | --- | --- | --- | --- |
| **Density, nb/min** | | | | | |
| Hippocampus | 65.9 | 54.2 | - | - | .34 |
| Hippocampus x Group | 7.9 | 63.4 | .000 | 0.02 | .95 |
| Insula | 1.8 | 29.4 | - | - | .97 |
| Insula x Group | 30.9 | 34.0 | .013 | 0.8 | .61 |
| Cingulate | 14.3 | 17.1 | - | - | .50 |
| Cingulate x Group | 25.6 | 20.1 | .023 | 1.6 | .45 |
| SMA | 15.4 | 35.9 | - | - | .76 |
| SMA x Group | 63.5 | 45.1 | .029 | 2.0 | .43 |
| Cerebellum | 0.3 | 5.2 | - | - | .97 |
| Cerebellum x Group | 15.9 | 6.3 | .079 | 6.4 | .11 |
| Heschl’s gyri | -38.5 | 175.9 | - | - | .90 |
| Heschl’s gyri x Group | 378.1 | 226.3 | .042 | 2.8 | .32 |
| Thalamus | 49.0 | 35.3 | - | - | .27 |
| Thalamus x Group | 68.8 | 47.5 | .027 | 2.1 | .41 |
| mPFC | 22.8 | 21.5 | - | - | .40 |
| mPFC x Group | 29.9 | 27.1 | .018 | 1.2 | .53 |
| Putamen | 1.1 | 44.4 | - | - | .98 |
| Putamen x Group | 82.0 | 51.4 | .037 | 2.5 | .33 |
| Pallidum | 31.2 | 294.6 | - | - | .95 |
| Pallidum x Group | 573.3 | 348.1 | .039 | 2.7 | .32 |
| **Amplitude, μV** | | | | | |
| Hippocampus | 5843.7 | 1726.3 | - | - | **.009*** |
| Hippocampus x Group | -3360.5 | 2019.6 | .030 | 2.8 | .32 |
| Insula | 2694.7 | 942.7 | - | - | **.025*** |
| Insula x Group | -1374.5 | 1092.1 | .018 | 1.6 | .45 |
| Cingulate | 1826.4 | 556.3 | - | - | **.011*** |
| Cingulate x Group | -554.0 | 653.1 | .008 | 0.7 | .64 |
| SMA | 2935.3 | 1216.4 | - | - | **.049*** |
| SMA x Group | -986.0 | 1527.7 | .005 | 0.4 | .76 |
| Cerebellum | 348.0 | 193.8 | - | - | .15 |
| Cerebellum x Group | -42.9 | 233.9 | .000 | 0.03 | .93 |
| Heschl’s gyri | 15251.0 | 5758.7 | - | - | **.038*** |
| Heschl’s gyri x Group | -3958.9 | 7409.6 | .003 | 0.3 | .76 |
| Thalamus | 4655.3 | 1133.7 | - | - | **.003*** |
| Thalamus x Group | -1559.7 | 1525.6 | .010 | 1.0 | .55 |
| mPFC | 2553.8 | 691.3 | - | - | **.008*** |
| mPFC x Group | -1199.7 | 872.9 | .020 | 1.9 | .44 |
| Putamen | 3386.0 | 1472.0 | - | - | *.060* |
| Putamen x Group | -407.6 | 1702.0 | .001 | 0.1 | .91 |
| Pallidum | 23537.4 | 9744.6 | - | - | **.049*** |
| Pallidum x Group | -2283.3 | 11514.9 | .000 | 0.04 | .93 |
| **Frequency, Hz** | | | | | |
| Hippocampus | 225.2 | 49.3 | - | - | **< .001*** |
| Hippocampus x Group | -239.9 | 57.7 | .229 | 17.3 | **.010*** |
| Insula | 76.8 | 29.0 | - | - | **.038*** |
| Insula x Group | -72.2 | 33.6 | .076 | 4.6 | .16 |
| Cingulate | 60.0 | 16.8 | - | - | **.008*** |
| Cingulate x Group | -62.1 | 19.8 | .147 | 9.9 | **.047*** |
| SMA | 125.8 | 34.9 | - | - | **.008*** |
| SMA x Group | -109.4 | 43.9 | .093 | 6.2 | .11 |
| Cerebellum | 14.3 | 5.8 | - | - | **.048*** |
| Cerebellum x Group | -14.7 | 7.0 | .072 | 4.4 | .17 |
| Heschl’s gyri | 452.1 | 177.7 | - | - | **.043*** |
| Heschl’s gyri x Group | -494.5 | 228.6 | .077 | 4.7 | .16 |
| Thalamus | 148.3 | 35.4 | - | - | **.003*** |
| Thalamus x Group | -122.9 | 47.7 | .092 | 6.7 | .11 |
| mPFC | 77.6 | 20.9 | - | - | **.008*** |
| mPFC x Group | -75.4 | 26.3 | .120 | 8.2 | *.075* |
| Putamen | 159.4 | 42.9 | - | - | **.008*** |
| Putamen x Group | -169.9 | 49.6 | .171 | 11.7 | **.040*** |
| Pallidum | 998.6 | 288.6 | - | - | **.009*** |
| Pallidum x Group | -1102.9 | 341.1 | .156 | 10.5 | **.047*** |
| **Duration, s** | | | | | |
| Hippocampus | -11.1 | 8.7 | - | - | .31 |
| Hippocampus x Group | 9.9 | 10.2 | .016 | 1.0 | .58 |
| Insula | -0.9 | 4.7 | - | - | .90 |
| Insula x Group | 1.2 | 5.4 | .001 | 0.05 | .92 |
| Cingulate | -2.7 | 2.8 | - | - | .45 |
| Cingulate x Group | 4.3 | 3.3 | .028 | 1.7 | .45 |
| SMA | -3.7 | 5.8 | - | - | .62 |
| SMA x Group | 7.4 | 7.3 | .017 | 1.0 | .55 |
| Cerebellum | -1.0 | 0.9 | - | - | .38 |
| Cerebellum x Group | 2.0 | 1.1 | .054 | 3.4 | .24 |
| Heschl’s gyri | -11.2 | 28.0 | - | - | .77 |
| Heschl’s gyri x Group | 45.1 | 36.0 | .026 | 1.6 | .45 |
| Thalamus | -5.1 | 6.1 | - | - | .50 |
| Thalamus x Group | 11.3 | 8.2 | .031 | 1.9 | .44 |
| mPFC | -3.7 | 3.5 | - | - | .40 |
| mPFC x Group | 5.8 | 4.4 | .029 | 1.7 | .45 |
| Putamen | -5.7 | 7.2 | - | - | .53 |
| Putamen x Group | 9.7 | 8.4 | .022 | 1.3 | .49 |
| Pallidum | -21.4 | 48.1 | - | - | .76 |
| Pallidum x Group | 58.6 | 56.8 | .018 | 1.1 | .55 |
| **Sigma power, μV^2^** | | | | | |
| Hippocampus | 4461.5 | 1535.9 | - | - | **.023*** |
| Hippocampus x Group | -2163.4 | 1796.8 | .017 | 1.4 | 0.47 |
| Insula | 2137.3 | 826.3 | - | - | **.042*** |
| Insula x Group | -844.1 | 957.2 | .009 | 0.8 | .63 |
| Cingulate | 1472.9 | 496.2 | - | - | **.021*** |
| Cingulate x Group | -450.6 | 582.6 | .007 | 0.6 | .68 |
| SMA | 2361.4 | 1080.1 | - | - | *.075* |
| SMA x Group | -992.8 | 1356.6 | .007 | 0.5 | .70 |
| Cerebellum | 277.6 | 172.8 | - | - | .20 |
| Cerebellum x Group | -72.6 | 208.5 | .002 | 0.1 | .87 |
| Heschl’s gyri | 12117.1 | 5138.7 | - | - | **.055** |
| Heschl’s gyri x Group | -3722.0 | 6611.9 | .004 | 0.3 | .76 |
| Thalamus | 3588.1 | 1047.4 | - | - | **.009*** |
| Thalamus x Group | -1356.5 | 1409.6 | .010 | 0.9 | .58 |
| mPFC | 2043.8 | 617.3 | - | - | **.011*** |
| mPFC x Group | -942.6 | 779.5 | .017 | 1.5 | .47 |
| Putamen | 2526.9 | 1294.3 | - | - | .11 |
| Putamen x Group | 153.2 | 1496.5 | .000 | 0.01 | .95 |
| Pallidum | 17303.9 | 8593.7 | - | - | .10 |
| Pallidum x Group | 1701.2 | 10154.9 | .000 | 0.03 | .93 |

Significant FDR-corrected *p*-values (*p* < .05) are depicted in bold with an asterisk. Non-significant FDR-corrected *p* values with a trend towards significance are shown in italics. SMA = supplementary motor area; mPFC = medial prefrontal cortex; *b* = unstandardized regression coefficient; *SE* = standard error; R^2^_change_ = change in coefficient of determination; FDR = false discovery rate.

**Supplementary Table 6** Moderation analyses for the relationships between GMVs in ROI set 2 and parietal spindle characteristics and sigma power

| Regions | *b* | *SE* | *R*^2^ _change_ | *F* _(1, 54)_ | *p* _FDR-corrected_ |
| --- | --- | --- | --- | --- | --- |
| **Density, nb/min** | | | | | |
| Hippocampus | -39.4 | 39.1 | - | - | .43 |
| Hippocampus x Group | 73.4 | 45.8 | .045 | 2.6 | .33 |
| Insula | -31.0 | 20.4 | - | - | .23 |
| Insula x Group | 50.7 | 23.6 | .078 | 4.6 | .16 |
| Cingulate | -16.9 | 12.6 | - | - | .28 |
| Cingulate x Group | 27.6 | 14.7 | .061 | 3.5 | .24 |
| SMA | -47.0 | 25.7 | - | - | .14 |
| SMA x Group | 67.6 | 32.3 | .075 | 4.4 | .17 |
| Cerebellum | -5.6 | 4.0 | - | - | .27 |
| Cerebellum x Group | 11.0 | 4.9 | .085 | 5.1 | .15 |
| Heschl’s gyri | -111.2 | 125.4 | - | - | .49 |
| Heschl’s gyri x Group | 298.6 | 161.3 | .059 | 3.4 | .24 |
| Thalamus | -4.3 | 27.4 | - | - | .93 |
| Thalamus x Group | 48.1 | 36.9 | .030 | 1.7 | .45 |
| mPFC | -13.2 | 15.5 | - | - | .50 |
| mPFC x Group | 39.3 | 19.6 | .069 | 4.0 | .18 |
| Putamen | -66.0 | 31.3 | - | - | .087 |
| Putamen x Group | 96.0 | 36.2 | .115 | 7.0 | .11 |
| Pallidum | -427.8 | 209.0 | - | - | .10 |
| Pallidum x Group | 630.6 | 247.0 | .108 | 6.5 | .11 |
| **Amplitude, μV** | | | | | |
| Hippocampus | 3587.9 | 1839.8 | - | - | .11 |
| Hippocampus x Group | -1207.2 | 2152.3 | .005 | 0.3 | .76 |
| Insula | 1171.4 | 1000.3 | - | - | .36 |
| Insula x Group | 25.5 | 1158.8 | .000 | 0.001 | .98 |
| Cingulate | 1065.2 | 603.7 | - | - | .15 |
| Cingulate x Group | -188.3 | 708.7 | .001 | 0.1 | .90 |
| SMA | 1602.7 | 1272.0 | - | - | .32 |
| SMA x Group | -98.1 | 1597.6 | .000 | 0.004 | .97 |
| Cerebellum | 130.0 | 200.6 | - | - | .62 |
| Cerebellum x Group | 129.6 | 242.1 | .005 | 0.3 | .76 |
| Heschl’s gyri | 5424.0 | 6187.8 | - | - | .49 |
| Heschl’s gyri x Group | 2191.1 | 7961.7 | .001 | 0.1 | .90 |
| Thalamus | 3222.6 | 1247.0 | - | - | **.042*** |
| Thalamus x Group | -614.4 | 1678.1 | .002 | 0.1 | .87 |
| mPFC | 1795.8 | 735.0 | - | - | **.048*** |
| mPFC x Group | -557.9 | 928.1 | .005 | 0.4 | .76 |
| Putamen | 2208.1 | 1525.6 | - | - | .26 |
| Putamen x Group | 478.9 | 1763.9 | .001 | 0.1 | .90 |
| Pallidum | 17936.1 | 10026.8 | - | - | .15 |
| Pallidum x Group | 1800.8 | 11848.3 | .000 | 0.02 | .94 |
| **Frequency, Hz** | | | | | |
| Hippocampus | 278.9 | 75.9 | - | - | **.008*** |
| Hippocampus x Group | -327.9 | 88.8 | .188 | 13.6 | **.025*** |
| Insula | 127.0 | 41.9 | - | - | **.019*** |
| Insula x Group | -116.0 | 48.6 | .086 | 5.7 | .12 |
| Cingulate | 86.0 | 24.9 | - | - | **.009*** |
| Cingulate x Group | -91.4 | 29.2 | .139 | 9.8 | **.047*** |
| SMA | 163.1 | 52.8 | - | - | **.016*** |
| SMA x Group | -139.5 | 66.3 | .066 | 4.4 | .17 |
| Cerebellum | 21.6 | 8.6 | - | - | **.044*** |
| Cerebellum x Group | -20.7 | 10.3 | .063 | 4.0 | .18 |
| Heschl’s gyri | 902.6 | 248.0 | - | - | **.008*** |
| Heschl’s gyri x Group | -951.0 | 319.1 | .125 | 8.9 | *.061* |
| Thalamus | 178.1 | 55.1 | - | - | **.012*** |
| Thalamus x Group | -178.1 | 74.2 | .085 | 5.8 | .12 |
| mPFC | 103.9 | 31.4 | - | - | **.011*** |
| mPFC x Group | -93.5 | 39.7 | .081 | 5.5 | .12 |
| Putamen | 194.0 | 65.7 | - | - | **.021*** |
| Putamen x Group | -212.4 | 76.0 | .117 | 7.8 | *.079* |
| Pallidum | 1110.0 | 444.6 | - | - | **.046*** |
| Pallidum x Group | -1301.7 | 525.3 | .096 | 6.1 | .11 |
| **Duration, s** | | | | | |
| Hippocampus | 11.5 | 10.7 | - | - | .40 |
| Hippocampus x Group | -9.0 | 12.5 | .009 | 0.5 | .70 |
| Insula | 1.9 | 5.7 | - | - | .82 |
| Insula x Group | -2.3 | 6.6 | .002 | 0.1 | .87 |
| Cingulate | 0.5 | 3.5 | - | - | .93 |
| Cingulate x Group | -0.1 | 4.1 | .000 | 0.001 | .98 |
| SMA | 3.3 | 7.2 | - | - | .75 |
| SMA x Group | -3.1 | 9.1 | .002 | 0.1 | .87 |
| Cerebellum | -0.8 | 1.1 | - | - | .59 |
| Cerebellum x Group | 1.2 | 1.4 | .012 | 0.7 | .64 |
| Heschl’s gyri | -14.9 | 35.0 | - | - | .76 |
| Heschl’s gyri x Group | 18.5 | 45.0 | .003 | 0.2 | .86 |
| Thalamus | 11.8 | 7.4 | - | - | .20 |
| Thalamus x Group | -5.4 | 9.9 | .005 | 0.3 | .76 |
| mPFC | 0.2 | 4.4 | - | - | .97 |
| mPFC x Group | -1.7 | 5.5 | .002 | 0.1 | .89 |
| Putamen | 12.5 | 8.8 | - | - | .26 |
| Putamen x Group | -8.5 | 10.2 | .011 | 0.7 | .64 |
| Pallidum | 122.9 | 57.3 | - | - | .081 |
| Pallidum x Group | -87.7 | 67.7 | .027 | 1.7 | .45 |
| **Sigma power, μV^2^** | | | | | |
| Hippocampus | 110.5 | 44.7 | - | - | **.048*** |
| Hippocampus x Group | -71.1 | 52.3 | .027 | 1.8 | .44 |
| Insula | 33.8 | 24.7 | - | - | .28 |
| Insula x Group | -18.1 | 28.6 | .006 | 0.4 | .76 |
| Cingulate | 34.1 | 14.6 | - | - | *.057* |
| Cingulate x Group | -17.6 | 17.1 | .016 | 1.1 | .55 |
| SMA | 62.8 | 30.6 | - | - | .10 |
| SMA x Group | -39.6 | 38.4 | .016 | 1.1 | .55 |
| Cerebellum | 7.1 | 4.9 | - | - | .25 |
| Cerebellum x Group | -2.3 | 5.9 | .002 | 0.2 | .87 |
| Heschl’s gyri | 135.7 | 152.3 | - | - | .49 |
| Heschl’s gyri x Group | -18.7 | 196.0 | .000 | 0.01 | .95 |
| Thalamus | 82.0 | 30.7 | - | - | **.038*** |
| Thalamus x Group | -30.9 | 41.4 | .008 | 0.6 | .69 |
| mPFC | 50.7 | 17.9 | - | - | **.026*** |
| mPFC x Group | -33.0 | 22.5 | .030 | 2.1 | .41 |
| Putamen | 81.2 | 36.9 | - | - | *.074* |
| Putamen x Group | -24.7 | 42.6 | .005 | 0.3 | .76 |
| Pallidum | 612.6 | 240.6 | - | - | **.043*** |
| Pallidum x Group | -176.0 | 284.4 | .005 | 0.4 | .76 |

Significant FDR-corrected *p*-values (*p* < .05) are depicted in bold with an asterisk. Non-significant FDR-corrected *p* values with a trend towards significance are shown in italics. SMA = supplementary motor area; mPFC = medial prefrontal cortex; *b* = unstandardized regression coefficient; *SE* = standard error; R^2^_change_ = change in coefficient of determination; FDR = false discovery rate.

**Supplementary Table 7** Moderation analyses for the relationships between GMVs in ROI set 1 and central spindle characteristics and sigma power, controlling for sleep and mood principal components in addition to age

| Regions | *b* | *SE* | *R*^2^ _change_ | *F* _change (1, 54)_ | *p* _FDR-corrected_ |
| --- | --- | --- | --- | --- | --- |
| **Density, nb/min** | | | | | |
| L. frontotemporal | -1.8 | 21.7 | - | - | .93 |
| L. frontotemporal x Group | 36.5 | 25.3 | .035 | 2.1 | .32 |
| R. frontotemporal | -19.0 | 29.2 | - | - | .65 |
| R. frontotemporal x Group | 45.9 | 33.0 | .034 | 1.9 | .32 |
| L. temporal | 25.6 | 140.8 | - | - | .92 |
| L. temporal x Group | 127.1 | 157.9 | .011 | 0.6 | .53 |
| **Amplitude, μV** | | | | | |
| L. frontotemporal | 2383.4 | 757.1 | - | - | **.010*** |
| L. frontotemporal x Group | -971.0 | 881.3 | .016 | 1.2 | 0.42 |
| R. frontotemporal | 2377.4 | 1050.9 | - | - | *.053* |
| R. frontotemporal x Group | -887.4 | 1187.3 | .008 | 0.6 | .53 |
| L. temporal | 16794.2 | 4861.6 | - | - | **.006*** |
| L. temporal x Group | -11288.2 | 5452.6 | .056 | 4.3 | .16 |
| **Frequency, Hz** | | | | | |
| L. frontotemporal | 103.1 | 26.1 | - | - | **.003*** |
| L. frontotemporal x Group | -108.5 | 30.4 | .171 | 12.7 | **.006*** |
| R. frontotemporal | 125.5 | 34.6 | - | - | **.005*** |
| R. frontotemporal x Group | -146.6 | 39.1 | .192 | 14.1 | **.006*** |
| L. temporal | 389.1 | 181.2 | - | - | *.061* |
| L. temporal x Group | -470.7 | 203.2 | .086 | 5.4 | .12 |
| **Duration, s** | | | | | |
| L. frontotemporal | 3.6 | 4.2 | - | - | .53 |
| L. frontotemporal x Group | -3.2 | 4.9 | .007 | 0.4 | .55 |
| R. frontotemporal | 1.5 | 5.5 | - | - | .92 |
| R. frontotemporal x Group | -2.9 | 6.2 | .004 | 0.2 | .64 |
| L. temporal | 23.8 | 26.6 | - | - | .53 |
| L. temporal x Group | -23.3 | 29.9 | .011 | 0.6 | .53 |
| **Sigma power, μV^2^** |  |  |  |  |  |
| L. frontotemporal | 57.5 | 18.5 | - | - | **.010*** |
| L. frontotemporal x Group | -31.2 | 21.6 | .026 | 2.1 | .32 |
| R. frontotemporal | 60.0 | 25.4 | - | - | **.047*** |
| R. frontotemporal x Group | -31.5 | 28.7 | .016 | 1.2 | .42 |
| L. temporal | 355.1 | 120.9 | - | - | **.013*** |
| L. temporal x Group | -262.9 | 135.6 | .049 | 3.8 | .17 |

FDR-corrected statistically significant *p*-values (*p* < .05) are depicted in bold with an asterisk. Non-significant FDR-corrected *p* values with a trend towards significance are shown in italics. *b* = unstandardized regression coefficient; *SE* = standard error; R^2^_change_ = change in coefficient of determination; FDR = false discovery rate; L. = left; R. = right.

**Supplementary Table 8** Moderation analyses for the relationships between GMVs in ROI set 2 and central spindle characteristics and sigma power, controlling for sleep and mood principal components in addition to age

| Regions | *b* | *SE* | *R*^2^ _change_ | *F* _(1, 54)_ | *p* _FDR-corrected_ |
| --- | --- | --- | --- | --- | --- |
| **Density, nb/min** | | | | | |
| Hippocampus | -7.0 | 48.0 | - | - | .92 |
| Hippocampus x Group | 72.8 | 55.9 | .029 | 1.7 | .47 |
| Insula | -23.3 | 25.5 | - | - | .52 |
| Insula x Group | 52.8 | 29.4 | .055 | 3.2 | .25 |
| Cingulate | -8.6 | 15.6 | - | - | .70 |
| Cingulate x Group | 32.5 | 18.2 | .054 | 3.2 | .25 |
| SMA | -26.5 | 32.6 | - | - | .54 |
| SMA x Group | 68.3 | 40.7 | .049 | 2.8 | .29 |
| Cerebellum | -4.3 | 4.9 | - | - | .54 |
| Cerebellum x Group | 14.7 | 5.8 | .100 | 6.4 | .080 |
| Heschl’s gyri | -116.5 | 153.3 | - | - | .56 |
| Heschl’s gyri x Group | 390.3 | 196.1 | .066 | 4.0 | .19 |
| Thalamus | 18.0 | 32.8 | - | - | .70 |
| Thalamus x Group | 68.6 | 43.5 | .039 | 2.5 | .34 |
| mPFC | -1.5 | 19.6 | - | - | .94 |
| mPFC x Group | 36.9 | 24.4 | .039 | 2.3 | .36 |
| Putamen | -49.1 | 39.1 | - | - | .37 |
| Putamen x Group | 106.9 | 44.7 | .093 | 5.7 | .093 |
| Pallidum | -298.4 | 260.1 | - | - | .39 |
| Pallidum x Group | 702.2 | 305.1 | .087 | 5.3 | .11 |
| **Amplitude, μV** | | | | | |
| Hippocampus | 3989.6 | 1749.1 | - | - | *.079* |
| Hippocampus x Group | -1642.9 | 2036.5 | .009 | 0.7 | .73 |
| Insula | 1625.3 | 946.0 | - | - | .18 |
| Insula x Group | -297.2 | 1091.8 | .001 | 0.1 | .90 |
| Cingulate | 1159.2 | 578.9 | - | - | .12 |
| Cingulate x Group | -170.8 | 672.1 | .001 | 0.1 | .90 |
| SMA | 1816.6 | 1223.6 | - | - | .27 |
| SMA x Group | -145.4 | 1529.5 | .000 | 0.0 | .94 |
| Cerebellum | 192.5 | 191.6 | - | - | .47 |
| Cerebellum x Group | 140.2 | 226.9 | .006 | 0.4 | .81 |
| Heschl’s gyri | 7175.0 | 5845.1 | - | - | .37 |
| Heschl’s gyri x Group | 1876.6 | 7479.8 | .001 | 0.1 | .90 |
| Thalamus | 3846.5 | 1172.2 | - | - | **.021*** |
| Thalamus x Group | -1296.7 | 1553.8 | .009 | 0.7 | .73 |
| mPFC | 1966.0 | 700.9 | - | - | **.032*** |
| mPFC x Group | -555.9 | 873.8 | .006 | 0.4 | .81 |
| Putamen | 2404.9 | 1449.5 | - | - | .20 |
| Putamen x Group | 427.4 | 1658.4 | .001 | 0.1 | .90 |
| Pallidum | 18116.8 | 9516.9 | - | - | .14 |
| Pallidum x Group | 2393.2 | 11165.6 | .001 | 0.0 | .90 |
| **Frequency, Hz** | | | | | |
| Hippocampus | 224.0 | 57.3 | - | - | **.015*** |
| Hippocampus x Group | -257.0 | 66.7 | .198 | 14.9 | **.015*** |
| Insula | 92.3 | 32.2 | - | - | **.031*** |
| Insula x Group | -96.1 | 37.2 | .101 | 6.7 | *.079* |
| Cingulate | 60.5 | 19.4 | - | - | **.021*** |
| Cingulate x Group | -72.2 | 22.5 | .148 | 10.3 | *.058* |
| SMA | 111.6 | 41.0 | - | - | **.037*** |
| SMA x Group | -124.6 | 51.3 | .090 | 5.9 | .093 |
| Cerebellum | 13.9 | 6.7 | - | - | .11 |
| Cerebellum x Group | -16.7 | 7.9 | .072 | 4.4 | .15 |
| Heschl’s gyri | 591.9 | 192.0 | - | - | **.021*** |
| Heschl’s gyri x Group | -730.4 | 245.7 | .129 | 8.8 | *.063* |
| Thalamus | 146.2 | 42.0 | - | - | **.021*** |
| Thalamus x Group | -151.4 | 55.7 | .105 | 7.4 | *.071* |
| mPFC | 75.6 | 24.3 | - | - | **.021*** |
| mPFC x Group | -85.2 | 30.3 | .116 | 7.9 | *.069* |
| Putamen | 149.3 | 50.1 | - | - | **.024*** |
| Putamen x Group | -168.3 | 57.4 | .128 | 8.6 | *.063* |
| Pallidum | 879.2 | 337.4 | - | - | **.046*** |
| Pallidum x Group | -1058.5 | 395.8 | .109 | 7.2 | *.071* |
| **Duration, s** | | | | | |
| Hippocampus | 4.4 | 9.2 | - | - | .72 |
| Hippocampus x Group | -6.5 | 10.8 | .006 | 0.4 | .81 |
| Insula | 0.9 | 4.9 | - | - | .92 |
| Insula x Group | -2.7 | 5.6 | .004 | 0.2 | .90 |
| Cingulate | -1.4 | 3.0 | - | - | .72 |
| Cingulate x Group | 0.5 | 3.5 | .000 | 0.0 | .92 |
| SMA | 0.8 | 6.2 | - | - | .92 |
| SMA x Group | -2.1 | 7.8 | .001 | 0.1 | .90 |
| Cerebellum | -0.8 | 1.0 | - | - | .54 |
| Cerebellum x Group | 1.2 | 1.2 | .018 | 1.0 | .67 |
| Heschl’s gyri | -24.2 | 29.5 | - | - | .54 |
| Heschl’s gyri x Group | 26.9 | 37.7 | .009 | 0.5 | .77 |
| Thalamus | 7.6 | 6.5 | - | - | .38 |
| Thalamus x Group | -3.5 | 8.6 | .003 | 0.2 | .90 |
| mPFC | -0.8 | 3.7 | - | - | .90 |
| mPFC x Group | -1.0 | 4.7 | .001 | 0.0 | .90 |
| Putamen | 3.5 | 7.7 | - | - | .72 |
| Putamen x Group | -3.1 | 8.8 | .002 | 0.1 | .90 |
| Pallidum | 61.2 | 50.3 | - | - | .37 |
| Pallidum x Group | -48.4 | 59.0 | .012 | 0.7 | .73 |
| **Sigma power, μV^2^** | | | | | |
| Hippocampus | 103.4 | 42.5 | - | - | *.058* |
| Hippocampus x Group | -65.3 | 49.4 | .024 | 1.7 | .47 |
| Insula | 41.8 | 23.0 | - | - | .16 |
| Insula x Group | -19.2 | 26.5 | .008 | 0.5 | .77 |
| Cingulate | 33.8 | 13.8 | - | - | *.058* |
| Cingulate x Group | -13.4 | 16.0 | .009 | 0.7 | .73 |
| SMA | 62.9 | 28.9 | - | - | .090 |
| SMA x Group | -29.1 | 36.1 | .009 | 0.7 | .73 |
| Cerebellum | 8.3 | 4.6 | - | - | .16 |
| Cerebellum x Group | -1.1 | 5.4 | .001 | 0.0 | .90 |
| Heschl’s gyri | 172.3 | 141.6 | - | - | .37 |
| Heschl’s gyri x Group | -2.2 | 181.2 | .000 | 0.0 | .99 |
| Thalamus | 89.1 | 28.9 | - | - | **.021*** |
| Thalamus x Group | -40.3 | 38.3 | .014 | 1.1 | .65 |
| mPFC | 52.1 | 16.8 | - | - | **.021*** |
| mPFC x Group | -26.5 | 20.9 | .021 | 1.6 | .48 |
| Putamen | 77.1 | 34.6 | - | - | .084 |
| Putamen x Group | -15.7 | 39.6 | .002 | 0.2 | .90 |
| Pallidum | 553.3 | 226.0 | - | - | *.058* |
| Pallidum x Group | -93.5 | 265.1 | .002 | 0.1 | .90 |

Significant FDR-corrected *p*-values (*p* < .05) are depicted in bold with an asterisk. Non-significant FDR-corrected *p* values with a trend towards significance are shown in italics. SMA = supplementary motor area; mPFC = medial prefrontal cortex; *b* = unstandardized regression coefficient; *SE* = standard error; R^2^_change_ = change in coefficient of determination; FDR = false discovery rate.
